# Associations Between Dietary Habits and Accelerated Aging and the Establishment of an Accelerated Aging Interpretable Risk Prediction Model via Shapley Additive Explanations: Cross-Sectional Study From Two Representative Populations

**DOI:** 10.2196/72020

**Published:** 2025-12-01

**Authors:** Zhengyang Wu, Ning Zhang, Haiwei Wang, Yang Yang, Houhao Shen, Qidi Zhang, Yunxia Cao, Yinan Du, Dongmei Ji

**Affiliations:** 1 Department of Obstetrics and Gynecology NHC Key Laboratory of Study on Abnormal Gametes and Reproductive Tract First Affliated Hospital of Anhui Medical University Hefei China; 2 Engineering Research Center of Biopreservation and Artificial Organs, Ministry of Education Hefei China; 3 Key Laboratory of Population Health Across Life Cycle (Anhui Medical University), Ministry of Education of the People’s Republic of China Hefei China; 4 School of Basic Medical Sciences Anhui Medical University Hefei China

**Keywords:** dietary indices, accelerated aging, machine learning, National Health and Nutrition Examination Survey, NHANES, UK Biobank

## Abstract

**Background:**

Research has revealed potential links between specific dietary habits and accelerated aging. However, most studies focus only on singular diets or lack ethnic diversity.

**Objective:**

This study aimed to investigate the associations between 5 dietary indices and the risk of accelerated aging and develop an interpretable machine learning (ML) model for accelerated aging prediction.

**Methods:**

We explored associations between dietary indices and the risk of accelerated aging using data from the US National Health and Nutrition Examination Survey (NHANES) and the UK Biobank. A weighted linear regression analysis was used to determine whether accelerated aging was linked to dietary habits, and the covariates were gradually adjusted to ensure that the association was stable. Nonlinear correlations were explored using restricted cubic spline curves. In addition, multiple ML algorithms were used to build predictive models of accelerated aging risk.

**Results:**

Except for the Dietary Inflammation Index (β=0.35, 95% CI 0.23-0.74), the other 4 dietary indices (Alternative Healthy Eating Index, Alternative Mediterranean Diet, Healthy Eating Index-2020, and Dietary Approaches to Stop Hypertension) were negatively associated with the risk of accelerated aging in NHANES participants. Similar results were observed in UK Biobank participants. Nine ML algorithms were used to develop risk prediction models, among which the gradient boosting decision tree model showed the best overall performance. A web-based prediction platform was developed and made publicly available.

**Conclusions:**

Significant associations between accelerated aging and dietary indices were observed. High compliance with the Dietary Inflammation Index had a promoting effect on accelerated aging, while high compliance with the Alternative Healthy Eating Index, Alternative Mediterranean Diet, Healthy Eating Index-2020, and Dietary Approaches to Stop Hypertension showed varying degrees of protection against accelerated aging.

## Introduction

### Biological Aging and Its Measurement

Aging is a highly complex biological process, typically attributed to molecular alterations and the accumulation of detrimental biomarkers that compromise tissue and organ function and regeneration, ultimately resulting in disease and mortality [1-3]. However, chronological age does not always accurately reflect biological aging [4]. Accelerated aging occurs when biological age exceeds chronological age. Ideally, the measurement of biological aging should reflect aging status across multiple levels of biological systems. Current studies have proposed several methods for measuring biological aging, ranging from individual biomarkers (such as telomere length) to integrated algorithms from epigenetic, proteomic, metabolomic, and other omics data [5,6]. However, these methods are prohibitively expensive for practical use, and their accuracy remains contentious. In this context, Klemera and Doubal [7] and Levine et al [8] proposed new biological age algorithms, the Klemera-Doubal method (KDM) and the PhenoAge algorithm, respectively. These algorithms have since been widely used for predicting biological age and are considered among the most accurate measures currently available [9,10].

### Dietary Patterns, Inflammation, and Accelerated Aging

Dietary habits directly influence the proportion of nutrients absorbed by the human body, thereby impacting endocrine processes and subsequently affecting normal metabolism. Several studies have shown that high-quality diets are associated with reduced risks of mortality, cancer, and cardiovascular disease [11-13]. Adherence to the typical Mediterranean diet was found to effectively reduce oxidative stress levels [12]. In addition, dietary indices such as the Dietary Approaches to Stop Hypertension (DASH), the Alternative Healthy Eating Index (AHEI), the Healthy Eating Index-2020 (HEI-2020), and the Dietary Inflammatory Index (DII) have been extensively studied and proven to effectively evaluate the healthiness of individuals’ dietary habits [14]. Recent research has also explored the relationship between dietary indices and accelerated aging. One study showed that inflammatory diets could significantly accelerate the aging process [15]. In addition, a large cohort study suggested that healthy diet habits might mitigate the adverse effects of pollutants on aging [16,17]. However, previous studies on the relationship between dietary indices and accelerated aging have had limitations, such as a focus on specific dietary indices or a lack of diversity in cohort demographics. These constraints highlight the need for further research using comprehensive datasets and diverse dietary indices to provide more robust and generalizable insights.

In this study, we simultaneously analyzed data from 2 representative populations in Europe and the United States to assess the relationship between dietary indices and accelerated aging. Weighted multivariable-adjusted linear regression was used to explore associations between 5 dietary indices and accelerated aging. Subset analyses were conducted to ensure the robustness of the results. In addition, we developed a machine learning (ML)–based web-based prediction tool for estimating accelerated aging risk based on individual dietary habits.

## Methods

### Study Population

The National Health and Nutrition Examination Survey (NHANES) is a nationally representative, population-based survey designed to assess the health and nutritional status of adults and children in the United States. Data from 7 consecutive survey cycles from 2005 to 2018 were included. We excluded participants younger than 20 years, as recommended by the NHANES for adult analyses. The UK Biobank is a prospective cohort study that enrolled individuals across the United Kingdom between 2006 and 2010 [18]. At baseline, participants completed questionnaires covering socioeconomic status, lifestyle, and health history, followed by a nurse-led interview to verify health information and perform standardized physical measurements. We analyzed cross-sectional data from the baseline assessment of the UK Biobank. Individuals were excluded if they lacked essential dietary intake data, blood biochemical measurements, or key covariates. We further excluded individuals with extreme energy intake (male individuals: <600 or >4800 kcal/d; female individuals: <500 or >3600 kcal/d) to minimize potential bias from aberrant energy consumption. A total of 10,940 eligible participants from the NHANES and 8594 participants from the UK Biobank were included in the study.

### Dietary Assessment and Calculation of Dietary Indices

We obtained detailed dietary intake information from NHANES participants to estimate energy, nutrient, and other food component consumption during the 24 hours preceding the interview. All participants were eligible for two 24-hour dietary recall interviews. The first interview was conducted in person at the NHANES mobile examination center, and the second was administered by telephone 3 to 10 days later. Only participants who completed both recalls were included in the analysis. Within the UK Biobank, participants recruited between 2006 and 2010 completed the Oxford WebQ dietary questionnaire during their baseline assessment visit. This questionnaire retrospectively assessed the consumption of up to 206 food items and 32 beverage types over the preceding 24 hours. The total intake of each food or beverage was calculated by multiplying the standard portion size by the reported frequency of consumption [19]. Only participants who completed at least 2 Oxford WebQ assessments were included in the analysis.

Dietary indices were calculated using the R package [20] *dietaryindex* [21]. This package can calculate various dietary indices of high interest in epidemiology, public health, nutrition, and clinical research, including the AHEI, DASH, HEI-2020, DII, and Alternative Mediterranean Diet (aMED). A detailed description of these dietary indices is provided in Multimedia Appendix 1 [22-24].

### Calculation of Accelerated Aging

KDM and PhenoAge were calculated using the R package *BioAge*. PhenoAge was calculated based on chronological age and 13 biomarkers. These biomarkers include albumin, alkaline phosphatase, blood urea nitrogen, creatinine, C-reactive protein, glycated hemoglobin, total cholesterol, uric acid, white blood cell count, lymphocyte percentage, mean cell volume, and red cell distribution width. PhenoAge represents an individual’s expected age, consistent with the estimated mortality risk based on their biochemical biomarker profile. KDM models biological age as the average biological state associated with a specific chronological age in the reference population, assuming that biological age increases linearly over time [25]. The biochemical indicators required by the KDM and PhenoAge algorithms are more easily obtainable than those required by traditional DNA methylation–based aging algorithms [9,26,27].

### Covariates

The survey data included demographic information, health status, and lifestyle factors, including age, sex (male or female), race (White, Black, or other), educational level, marital status, deprivation index, family size, BMI, history of hypertension, physical activity (PA), smoking history, alcohol consumption, and daily energy intake [28]. For NHANES participants, the deprivation index was categorized based on the poverty income ratio (PIR) as higher (most deprived; PIR <1.5), middle (3.5>PIR≥1.5), and lower (least deprived; PIR ≥3.5) [29]. PA was measured on the basis of the Global Physical Activity Questionnaire, which includes questions related to work-related, leisure-time, and sedentary activities. In the UK Biobank, participants’ deprivation index was calculated based on the Townsend Deprivation Index, which was constructed from 4 key variables: unemployment, household overcrowding, lack of car ownership, and lack of home ownership. A higher index indicated a higher level of deprivation. PA was estimated as metabolic equivalent task (MET) minutes using items derived from the International Physical Activity Questionnaire [30]. For participants from both the NHANES and the UK Biobank, PA levels were classified according to MET minutes per week into 3 categories: inactive (≤499 MET-min/wk), moderately active (500-1554 MET-min/wk), and regular exercisers (≥1555 MET-min/wk) [25]. Observations with missing data for any covariate were excluded from the analysis.

### Development of ML Models

Nine ML algorithms were used for estimating accelerated aging risk: gradient boosting decision tree (GBDT), random forest, decision tree, tabular prior-data fitted networks, logistic regression, light gradient boosting machine, categorical boosting, deep learning, and extreme gradient boosting based on the area under the receiver operating characteristic curve (AUROC) and the area under the precision-recall curve (AUPRC). We used the NHANES dataset as the training set for the model. Multivariate factors such as demographics, lifestyle, disease history, and dietary indices were used to predict accelerated aging risk. To validate the predictive effect, we used 70% of the data for model training and 30% for testing. To assess model stability and prediction accuracy, we conducted 5-fold cross-validation on the training set. Owing to imbalanced data distribution, we used synthetic minority oversampling technique for balancing. In addition to internal cross-validation, we included baseline participants from the UK Biobank dataset as external validation data. To apply the established early aging risk prediction model, a web-based application was developed using the Python *Streamlit* library.

### Statistical Analysis

Group comparisons between participant characteristics and accelerated aging values were conducted using 2-tailed *t* tests for continuous variables and chi-square or Fisher exact tests for categorical variables, as appropriate. Participants’ characteristics were presented as means and SDs for continuous variables and as percentages for categorical variables. Subsequently, the relationships between the 5 dietary indices (HEI-2020, aMED, DII, DASH, and AHEI) and accelerated aging (calculated by the PhenoAge and KDM algorithms) were assessed using linear regression, with adjustments for covariates. BMI, history of hypertension, educational level, and smoking history from the covariates were considered stratification variables to stratify participants. Further regression analysis was conducted to determine the impact of diet on accelerated aging across different strata. All analyses involving NHANES data in this study used survey weights for weighted analysis, balancing the unequal selection probabilities arising from the NHANES sampling survey design, to obtain nationally representative estimates. The study followed the STROBE (Strengthening the Reporting of Observational Studies in Epidemiology) reporting guideline. All data analyses and modeling processes were completed using Stata (version 17.0; StataCorp LLC), Python (version 3.10; Python Software Foundation), or R (version 4.3.0; R Foundation for Statistical Computing). Statistical significance was set at 2-tailed *P*<.05.

### Ethical Considerations

NHANES investigators obtained appropriate ethical approvals for the publicly available data. The study was approved by the National Center for Health Statistics Ethics Review Board (Protocols: #2005-06, Continuation of Protocol #2005-06, #2011-17, Continuation of Protocol #2011-17, and #2018-01). The research was conducted in accordance with the Declaration of Helsinki. Informed consent was obtained by NHANES investigators from all participants involved in the research. Furthermore, the UK Biobank received ethics approval from the North West Multicenter Research Ethics Committee. Our study used UK Biobank resources (application 211452).

## Results

### Participant Characteristics

**Figure 1:**
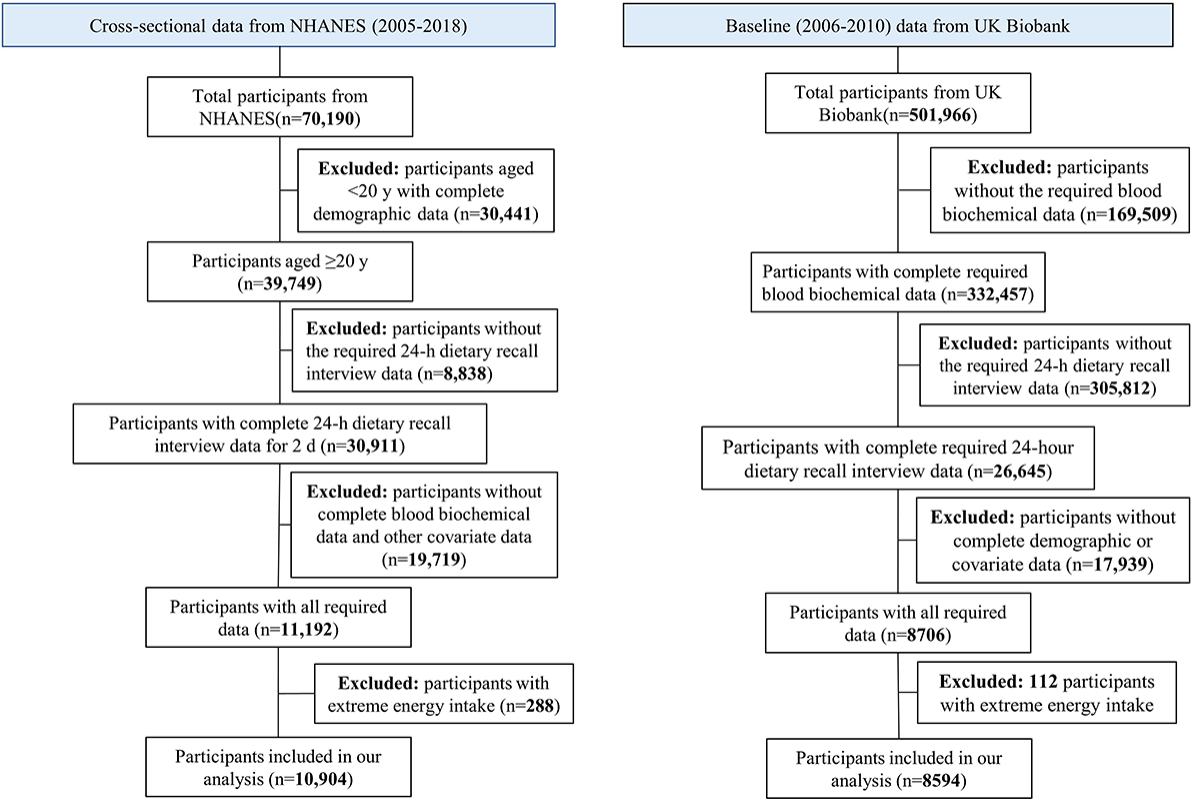
Participant selection flow chart.

From the NHANES population, 10,904 participants, representing an estimated 476,381,485 American adults, were included (Figure 1). The overall characteristics of the participants are summarized in Table 1. Their average age was 48.61 (SD 17.40) years; 52.49% (5724/10,904) were female, 72.48% (7903/10,904) were White, and 79.9% (8712/10,904) had an educational level higher than high school. The mean age acceleration was –5.23 (SD 14.56) years, indicating that participants’ KDM age was on average 5.23 years lower than their chronological age. Moreover, the mean senescence time for accelerated aging calculated with PhenoAge was −0.85 (SD 6.45) years. The scores for the 5 dietary indices approximately followed normal distributions (Figure S1 in Multimedia Appendix 1).

**Table 1:** Demographic characteristics of US National Health and Nutrition Examination Survey participants.

Characteristics		Overall (n=10,904)	KDM^a^ age			*P* value^b^		PhenoAge					*P* value^b^
			Accelerated aging (n=7258)	Nonaccelerated aging (n=3646)			Accelerated aging (n=6847)			Nonaccelerated aging (n=4057)			
Age (years), mean (SD)		48.61 (17.40)	51.09 (16.92)	43.68 (17.29)	*<.001* ^c^		47.58 (17.11)			50.35 (17.74)		*<.001*	
**Sex, n (%)**						.84							*<.001*
	Female	5724 (52.49)	3805 (52.42)	1919 (52.63)			3821 (55.81)			1903 (46.91)			
	Male	5180 (47.51)	3453 (47.58)	1727 (47.37)			3026 (44.19)			2154 (53.09)			
**Race, n (%)**						*<.001*							*<.001*
	Black	2029 (18.61)	1150 (15.84)	879 (24.11)			1031 (15.06)			998 (24.6)			
	White	7903 (72.48)	5458 (75.2)	2445 (67.06)			5203 (75.99)			2700 (66.55)			
	Other	972 (8.91)	650 (8.96)	322 (8.83)			613 (8.95)			359 (8.85)			
**Education, n (%)**						*<.001*							*.002*
	High school graduate or GED^d^ or equivalent	1318 (12.09)	822 (11.33)	496 (13.6)			769 (11.23)			549 (13.53)			
	Less than high school	874 (8.02)	633 (8.72)	241 (6.61)			558 (8.15)			316 (7.79)			
	More than high school	8712 (79.9)	5803 (79.95)	2909 (79.79)			5520 (80.62)			3192 (78.68)			
**Marital status, n (%)**						*<.001*							*<.001*
	Divorced or living alone	4194 (38.46)	2594 (35.74)	1600 (43.88)			2470 (36.07)			1724 (42.49)			
	Other	6710 (61.54)	4664 (64.26)	2046 (56.12)			4377 (63.93)			2333 (57.51)			
**Deprivation Index, n (%)**						*<.001*							*<.001*
	Higher (most deprived)	3686 (33.8)	2657 (36.61)	1029 (28.22)			2555 (37.32)			1131 (27.88)			
	Middle	3689 (33.83)	2423 (33.38)	1266 (34.72)			2272 (33.18)			1417 (34.93)			
	Lower (least deprived)	3529 (32.36)	2178 (30.01)	1351 (37.05)			2020 (29.5)			1509 (37.19)			
**Family size, n (%)**						*<.001*							.06
	1-3	6914 (63.41)	4728 (65.14)	2186 (59.96)			4287 (62.61)			2627 (64.75)			
	4-5	2923 (26.81)	1862 (25.65)	1061 (29.1)			1887 (27.56)			1036 (25.54)			
	≥6	1067 (9.79)	668 (9.2)	399 (10.94)			673 (9.83)			394 (9.71)			
**BMI (kg/m^2^, n (%)**						*<.001*							*<.001*
	<20	447 (4.1)	355 (4.89)	92 (2.52)			334 (4.88)			113 (2.79)			
	20-25	2550 (23.39)	1922 (26.48)	628 (17.22)			1896 (27.69)			654 (16.12)			
	>25	7907 (72.51)	4981 (68.63)	2926 (80.25)			4617 (67.43)			3290 (81.09)			
**Hypertension, n (%)**						*<.001*							*<.001*
	No	7221 (66.22)	5014 (69.08)	2207 (60.53)			4939 (72.13)			2282 (56.25)			
	Yes	3683 (33.78)	2244 (30.92)	1439 (39.47)			1908 (27.87)			1775 (43.75)			
**Physical activity, n (%)**						*<.001*							*<.001*
	Exercise regularly	5099 (46.76)	3242 (44.67)	1857 (50.93)			3267 (47.71)			1832 (45.16)			
	Moderately active	1930 (17.7)	1365 (18.81)	565 (15.5)			1289 (18.83)			641 (15.8)			
	Inactive	3875 (35.54)	2651 (36.53)	1224 (33.57)			2291 (33.46)			1584 (39.04)			
**Smoking status, n (%)**						*<.001*							*<.001*
	Current smoker	2142 (19.64)	1297 (17.87)	845 (23.18)			1097 (16.02)			1045 (25.76)			
	Ever smoker	2598 (23.83)	1831 (25.23)	767 (21.04)			1595 (23.29)			1003 (24.72)			
	Never smoker	6164 (56.53)	4130 (56.9)	2034 (55.79)			4155 (60.68)			2009 (49.52)			
**Alcohol consumption, n (%)**						*<.001*							*.049*
	No	1481 (13.58)	1044 (14.38)	437 (11.99)			964 (14.08)			517 (12.74)			
	Yes	9423 (86.42)	6214 (85.62)	3209 (88.01)			5883 (85.92)			3540 (87.26)			
Daily energy intake (kcal), mean (SD)		2010.68 (708.38)	2003.76 (701.24)	2024.44 (722.28)	.17		2011.27 (699.13)			2009.67 (723.81)		.80	
PhenoAge (years), mean (SD)		−0.85 (6.45)	−2.33 (5.63)	2.09 (6.96)	*<.001*		−4.66 (3.00)			5.59 (5.53)		*<.001*	
KDM age (years), mean (SD)		−5.23 (14.56)	−13.05 (9.22)	10.36 (10.01)	*<.001*		−8.90 (12.35)			0.97 (15.85)		*<.001*	
AHEI^e^ scores, mean (SD)		38.82 (11.63)	40.00 (11.75)	36.46 (11.02)	*<.001*		40.06 (11.69)			36.72 (11.22)		*<.001*	
aMED^f^ scores, mean (SD)		3.51 (1.38)	3.65 (1.39)	3.23 (1.33)	*<.001*		3.67 (1.38)			3.23 (1.34)		*<.001*	
DASH^g^ scores, mean (SD)		22.49 (5.06)	23.08 (5.07)	21.31 (4.84)	*<.001*		23.17 (5.03)			21.34 (4.90)		*<.001*	
DII^h^ scores, mean (SD)		1.15 (1.68)	1.04 (1.71)	1.37 (1.60)	*<.001*		1.01 (1.71)			1.38 (1.62)		*<.001*	
HEI-2020^i^ scores, mean (SD)		51.58 (11.97)	52.77 (11.97)	49.20 (11.61)	*<.001*		52.83 (11.92)			49.46 (11.75)		*<.001*	

^a^KDM: Klemera-Doubal method.

^b^Wilcoxon rank sum test for continuous variables; Pearson chi-square test for categorical variables.

^c^Italicization indicates values that met the threshold for significance (*P*<.05).

^d^GED: General Educational Development Test.

^e^AHEI: Alternative Healthy Eating Index.

^f^aMED: Alternative Mediterranean Diet.

^g^DASH: Dietary Approaches to Stop Hypertension.

^h^DII: Dietary Inflammatory Index.

^i^HEI-2020: Healthy Eating Index-2020.

From the UK Biobank cohort, 8594 participants were included, of whom 54.56% (n=4689) were female, 97.68% (n=8395) had White European ancestry, and 64.96% (n=8594) had attained higher education (Table S1 in Multimedia Appendix 1). The participants’ PhenoAge scores were on average 5.47 years lower than their chronological age, whereas the average age acceleration derived from KDM age was 17.16 years. Compared with the NHANES participants, those in the UK Biobank were older, had higher educational attainment, and exhibited lower BMI levels.

In addition, we noticed an overall positive correlation among the DASH, AHEI, HEI-2020, and aMED scores, except for the DII scores, which exhibited negative correlations with other indices, in both the NHANES and the UK Biobank. Among the NHANES participants, the strongest association was between the DASH and AHEI scores (weighted Pearson *r*=0.85), whereas among the UK Biobank participants, the strongest association was between the DII and AHEI scores (Pearson *r*=−0.78; Figure S2 in Multimedia Appendix 1).

### Correlations Between Different Dietary Indices and Accelerated Aging

For the analysis of NHANES participants, we constructed 3 weighted linear regression models and adjusted the confounders to find the correlations between different dietary indices and accelerated aging. Model 1 was only adjusted for age, sex, and race; model 2 was adjusted for model 1’s covariates, family size, educational level, deprivation index, and marital status; and model 3 was adjusted for model 2’s covariates, BMI, smoking status, alcohol consumption, PA, daily energy intake, and history of hypertension.

In model 1, the AHEI, DASH, aMED, and HEI-2020 scores were negatively correlated with accelerated aging calculated by both PhenoAge and KDM, whereas the DII scores were positively correlated with them (DII score for PhenoAge: β=0.35, 95% CI 0.23 to 0.46; DII score for KDM: β=0.55, 95% CI 0.36 to 0.74; Table 2). In models 2 and 3, the associations between the dietary indices and accelerated aging calculated by both PhenoAge and KDM remained significant (*P*<.05; Table 2). These results revealed correlations between the different dietary indices and accelerated aging.

**Table 2:** Weighted linear regression analysis of dietary indices and accelerated aging among US National Health and Nutrition Examination Survey participants.

Dietary indices	Model 1^a^					Model 2^b^					Model 3^c^			
	PhenoAge		KDM^d^ age		PhenoAge			KDM age		PhenoAge			KDM age	
	β (95% CI)	*P* value	β (95% CI)	*P* value	β (95% CI)		*P* value	β (95% CI)	*P* value	β (95% CI)		*P* value	β (95% CI)	*P* value
AHEI^e^	–0.09 (–0.11 to –0.07)	<.001	–0.16 (–0.19 to –0.13)	<.001	–0.08 (–0.09 to –0.06)		<.001	–0.15 (–0.18 to –0.12)	<.001	–0.06 (–0.08 to –0.04)		<.001	–0.11 (–0.13 to –0.08)	<.001
aMED^c^	–0.83 (–0.96 to –0.69)	<.001	–1.51 (–1.71 to –1.31)	<.001	–0.74 (–0.87 to –0.61)		<.001	–1.43 (–1.65 to –1.22)	<.001	–0.57 (–0.70 to –0.44)		<.001	–1.07 (–1.28 to –0.86)	<.001
DASH^d^	–0.24 (–0.27 to –0.20)	<.001	–0.44 (–0.50 to –0.38)	<.001	–0.21 (–0.25 to –0.18)		<.001	–0.42 (–0.49 to –0.36)	<.001	–0.16 (–0.20 to –0.13)		<.001	–0.32 (–0.38 to –0.26)	<.001
DII^e^	.53 (0.41 to 0.64)	<.001	.84 (0.65 to 1.03)	<.001	.45 (0.34 to 0.57)		<.001	.76 (0.56 to 0.96)	<.001	.35 (0.23 to 0.46)		<.001	.55 (0.36 to 0.74)	<.001
HEI-2020^f^	–0.09 (–0.10 to –0.07)	<.001	–0.15 (–0.17 to –0.13)	<.001	–0.08 (–0.10 to –0.06)		<.001	–0.14 (–0.17 to –0.12)	<.001	–0.06 (–0.08 to –0.05)		<.001	–0.10 (–0.13 to –0.08)	<.001

^a^Adjusted for age, sex, and race.

^b^Model 1+adjusted for family size, educational level, deprivation index, and marital status.

^c^Model 2+adjusted for BMI, smoking status, alcohol consumption, physical activity, daily energy intake, and history of hypertension.

^d^KDM: Klemera-Doubal method.

^e^AHEI: Alternative Healthy Eating Index.

^f^aMED: Alternative Mediterranean Diet.

^g^DASH: Dietary Approaches to Stop Hypertension.

^h^DII: Dietary Inflammatory Index.

^i^HEI-2020: Healthy Eating Index-2020.

In the analysis of UK Biobank participants too, we conducted linear regression analysis using 3 models, with covariate adjustments identical to those applied in the NHANES analyses. The results showed that the associations of AHEI, DASH, aMED, and DII scores with age acceleration were consistent with those observed in the NHANES analyses, whereas HEI-2020 scores showed no significant association with age acceleration in any of the 3 models (DII score for PhenoAge: *P*=.85; DII score for KDM: *P*=.83; Table S2 in Multimedia Appendix 1). In the fully adjusted model 3, higher aMED scores showed the strongest protective association with age acceleration, regardless of whether the KDM or PhenoAge algorithm was used (aMED score for PhenoAge: β=−0.08, 95% CI −0.15 to −0.01; aMED score for KDM: β=−0.39, 95% CI −0.55 to −0.23; Table S2 in Multimedia Appendix 1).

### Correlations Between Quartiles of 5 Dietary Indices and Accelerated Aging

In NHANES participants, linear regression analysis across quartiles of each of the 5 dietary indices showed significant correlations with accelerated aging, as calculated by both PhenoAge and KDM, with DII scores being positively correlated and the scores of the other 4 dietary indices being negatively correlated (Table 3). All correlations exhibited strong linear trends (*P* for trend<.001). We observed similar results in the analysis of UK Biobank participants, but the association between HEI-2020 scores and age acceleration was not significant (Table S3 in Multimedia Appendix 1).

**Table 3:** Quartile regression and linear trend analysis among US National Health and Nutrition Examination Survey participants.

Dietary indices and variables		PhenoAge			KDM^a^ age	
Characteristic		β (95% CI)	*P* value	β (95% CI)		*P* value
**AHEI^b^**						
	Q1	Reference	—^c^	Reference		—
	Q2	–0.74 (–1.10 to –0.37)	<.001^d^	–2.24 (–2.94 to –1.55)		<.001
	Q3	–1.04 (–1.42 to –0.67)	<.001	–3.62 (–4.64 to –2.60)		<.001
	Q4	–1.52 (–2.04 to –1.00)	<.001	–5.42 (–6.37 to –4.47)		<.001
	*P* for linear trend	—	<.001	—		<.001
**aMED^e^**						
	Q1	Reference	—	Reference		—
	Q2	–0.22 (–0.61 to 0.16)	.232	–1.27 (–2.33 to –0.20)		<.001
	Q3	–0.67 (–1.09 to –0.24)	.002	–3.79 (–4.88 to –2.70)		<.001
	Q4	–0.99 (–1.52 to –0.46)	<.001	–5.04 (–6.03 to –4.05)		<.001
	*P* for linear trend	—	<.001	—		<.001
**DASH^d^**						
	Q1	Reference	—	Reference		—
	Q2	–0.99 (–1.43 to –0.55)	<.001	–2.80 (–3.88 to –1.73)		<.001
	Q3	–1.23 (–1.63 to –0.82)	<.001	–4.09 (–4.86 to –3.31)		<.001
	Q4	–1.73 (–2.24 to –1.23)	<.001	–6.76 (–7.72 to –5.80)		<.001
	*P* for linear trend	—	<.001	—		<.001
**HEI-2020^e^**						
	Q1	Reference	—	Reference		—
	Q2	–0.75 (–1.08 to –0.42)	<.001	–2.08 (–2.98 to –1.17)		<.001
	Q3	–1.14 (–1.59 to –0.69)	<.001	–3.60 (–4.52 to –2.67)		<.001
	Q4	–1.5 (–2.00 to –0.99)	<.001	–5.13 (–6.11 to –4.14)		<.001
	*P* for linear trend	—	<.001	—		<.001
**DII^f^**						
	Q1	Reference	—	Reference		—
	Q2	0.44 (0.07 to 0.80)	.012	1.57 (0.69 to 2.45)		.003
	Q3	0.83 (0.33 to 1.32)	<.001	3.09 (2.11 to 4.06)		<.001
	Q4	1.11 (0.55 to 1.67)	<.001	2.26 (1.38 to 3.15)		<.001
	*P* for linear trend	—	<.001	—		<.001

^a^KDM: Klemera-Doubal method.

^b^AHEI: Alternative Healthy Eating Index.

^c^Not applicable.

^d^Italicization indicates values that met the threshold for significance (*P*<.05).

^e^aMED: Alternative Mediterranean Diet.

^f^DASH: Dietary Approaches to Stop Hypertension.

^g^HEI-2020: Healthy Eating Index-2020.

^h^DII: Dietary Inflammatory Index.

### Subgroup Analysis

To ascertain whether the association between accelerated aging and dietary indices was consistent across the general population, we conducted analyses stratified by BMI, history of hypertension, educational level, and smoking history. First, we performed the subgroup analyses in people with different BMI levels, examining the relationships between the dietary indices and accelerated aging calculated by both PhenoAge and KDM in NHANES participants. In the analysis of KDM age acceleration, higher aMED and DASH scores exhibited stronger protective effects in individuals who were obese (*P* for interaction aMED=.02; *P* for interaction DASH=.01), a finding that is consistent with the results from the UK Biobank analysis (interaction aMED: *P*=.002; interaction DASH: *P*=.03; Figure S3 in Multimedia Appendix 1). Subgroup analyses across other stratifications revealed no significant between-group heterogeneity (Figures S4-S10 in Multimedia Appendix 1).

### Nonlinear Relationship and Inflection Point in the Effects of Dietary Indices on Accelerated Aging

We used restricted cubic spline curves to explore the relationships between accelerated aging and the dietary indices. Our results remained unchanged after both primary and additional adjustments for potential confounders; therefore, only the most adjusted model (model 3) is reported. In the analysis of NHANES participants, we observed highly significant linear trends in the associations between the dietary indices and accelerated aging, quantified using both PhenoAge and KDM (overall association: *P*<.001; nonlinearity: *P*>.05). We observed similar results among participants in the UK Biobank, except that HEI-2020 scores showed no significant linear association with the 2 accelerated aging metrics in the UK Biobank analysis (Figures S11-S12 in Multimedia Appendix 1).

### Testing the Performance of ML Models in Identifying Accelerated Aging

From the previous analysis, we determined that there was a significant correlation between dietary indices and accelerated aging. We constructed an ML model and used Shapley additive explanations (SHAP) values to elucidate the relative importance of key predictive features. The NHANES data were split into training and testing sets in a 0.7 ratio, while the UK Biobank data were used as an external validation cohort. Model performance was quantified using the area under the receiver operating characteristic curve, while the area under the precision-recall curve characterized the precision-recall trade-off across classification thresholds. We evaluated 9 ML algorithms, including GBDT; categorical boosting achieved the highest AUROC (0.729) in the training set, whereas tabular prior-data fitted networks yielded the best performance (AUROC=0.757) in the test set (Figure S13 in Multimedia Appendix 1; Table S4 in Multimedia Appendix 1). However, to prioritize the stability, generalization, and interpretability of the model, we chose GBDT as the final modeling algorithm. This algorithm performed stably and well in both the training and test sets. The precision-recall curve (AUPRC=0.976) for the test set indicated very good prediction performance on imbalanced samples (Figure S14 in Multimedia Appendix 1). Specifically, the learning rate of this model was set to 0.1 to balance convergence and generalization. The maximum number of iterations of the learner was 58, and the maximum tree depth was 4 to constrain model complexity. The minimum segmentation requirement for each internal node was 50 samples to reduce overfitting. All experiments were conducted with fixed random seeds to ensure reproducibility. Furthermore, we evaluated the inclusion of different dietary indices to assess their influence on model performance. The hyperparameter information of other models is presented in Table S5 in Multimedia Appendix 1. The results demonstrated that the inclusion of the AHEI enhanced model performance in both the training (AUROC: 0.711 vs 0.682) and test (AUROC: 0.706 vs 0.680) sets. Incorporating aMED, DII, DASH, or HEI-2020 individually improved training set performance but compromised the model’s generalizability (Figure S15 in Multimedia Appendix 1). A web-based platform was developed and can be accessed [31].

### Interpretation of Personalized Predictions

The SHAP plot reveals the effect of each variable in the model on identifying accelerated aging cases within the test dataset. The honeycomb diagram (Figure S16A in Multimedia Appendix 1) arranges data points horizontally to visualize feature value distributions and their corresponding contributions to model predictions. Points positioned to the right reflect increased positive contributions to the prediction, whereas those to the left indicate stronger negative influences. In the feature importance bar chart (Figure S16B in Multimedia Appendix 1), the bar length is proportional to the relative importance of each feature. The analysis identified age, marital status, and AHEI score as the 3 most influential features in the model. In addition, force plots were generated separately for participants with accelerated aging and those without (Figures S16C and S16D in Multimedia Appendix 1). The bold value denotes the model’s predicted log-odds, f(x), for an individual, whereas the base value represents the expected prediction in the absence of any feature inputs; f(x) corresponds to the log-odds of accelerated aging for each observation. Features shown in red increase the predicted risk of accelerated aging, whereas those shown in blue decrease it. Arrow length visually encodes the magnitude of each feature’s contribution: longer arrows indicate stronger effects on the prediction.

## Discussion

Previous research has explored the associations between various dietary patterns and diseases such as cancer, hypertension, and Alzheimer disease [27,32,33]. This study found that 5 dietary indices had differing impacts on the acceleration of aging in the NHANES cohorts. DII scores were positively correlated with accelerated aging, while the AHEI, aMED, HEI-2020 and DASH scores were negatively correlated. Furthermore, we rigorously evaluated the final model’s performance using training, internal validation, and external validation cohorts comprising diverse populations from the United States and Europe. Integrated analyses of ROC and precision-recall curves unequivocally demonstrated the model’s superior predictive accuracy.

Diet is a crucial and easily modifiable risk factor for various chronic diseases [34]. Increasing evidence suggests that different dietary indices impact aging to varying degrees. The aMED and the DASH diet have been shown to have anti-inflammatory and cardioprotective effects, which may contribute to preventing dementia associated with neuroinflammation [35,36]. Our cross-sectional findings further suggest that a healthier dietary pattern could significantly reduce the risk of accelerated aging. Diet can regulate the immune system to some extent. Evidence suggests that the intake of certain nutrients and bioactive components can affect the process of neuroinflammation in animals, thereby affecting aging [37]. In addition, long-term consumption of a proinflammatory diet may reduce gut microbiota diversity, particularly decreasing the abundance of butyrate-producing *Firmicutes* [37,38]. Furthermore, interactions between the gut microbiota and the host immune system may result in prolonged low-grade inflammatory exposure in the host, thereby promoting the development of accelerated aging [39]. This finding is further corroborated by the positive association between the DII scores and an increased risk of accelerated aging observed in our study.

An important part of healthy aging involves screening for nutritional intake as well as developing and improving lifestyle intervention measures to support health behavior changes focused on aging in different populations [40]. In addition to dietary intake, BMI is considered an important indicator of health in older adults, with obesity being a major risk factor for unhealthy aging [41]. In subgroup analyses, we also observed that maintaining a good diet was more protective against the risk of accelerated aging in individuals with higher levels of obesity.

Subgroup analyses revealed that individuals with higher degrees of obesity exhibited increased susceptibility to both the age-accelerating effects of high DII eating patterns and the age-mitigating impacts of elevated DASH, aMED, HEI-2020, and AHEI scores. We hypothesize that this heightened sensitivity may stem from the generally less healthful dietary habits associated with obesity [42]. Interestingly, similar trends (*P* values for all 5 dietary indices for both PhenoAge and KDM age were <.05) were observed among nonsmokers and participants with lower educational attainment.

Currently, no widely recognized predictive model exists for accelerated aging. Previous studies have often focused on the predictive power of accelerated aging for common chronic diseases [43-45]. Among existing models, the one developed by Liu et al [46], based on free tri-iodothyronine, performs best, but it requires the collection of free tri-iodothyronine data from participants, which increases costs and hinders widespread adoption of the model. Several other predictive models have limited sample sizes, lack external validation, and exhibit AUROC values lower than those of our model [47,48]. The predictive model developed in this study integrates dietary indices and common demographic data; it achieves high prediction accuracy and enables data collection via questionnaires, demonstrating strong clinical feasibility. From a clinical application perspective, this web-based tool can be widely adopted in community primary care settings, allowing health care providers to rapidly perform initial screening for accelerated aging risk among residents or individuals undergoing routine health examinations. Especially during routine community health check-ups, medical staff can conveniently and quickly identify individual risk factors based on SHAP values provided by the web-based tool and offer corresponding recommendations.

The first strength of our study is that we included multiple biological age and dietary indices for comparison and incorporated sociological factors to stratify the population for further analysis among different groups, which verified the stability of the research results [15]. This study helps to further establish the connection between dietary health and accelerated aging, linking an essential factor of daily life, diet, with aging, and provides effective dietary adjustment strategies for aging prevention. In addition, many covariates related to lifestyle habits or personal health status were considered in our study, such as BMI, smoking history, history of hypertension, and PA.

Our study also has some limitations. First, the early aging risk prediction model developed in this study exhibited relatively poor predictive efficiency for positive results in the confusion matrix analysis due to a certain degree of bias in the training samples. Second, this is a cross-sectional study and could demonstrate an association between dietary habits and accelerated aging, but it could not provide evidence of causation. Further research is needed to elucidate the association and underlying mechanisms between eating habits and accelerated aging. Moreover, the cross-sectional nature of this study inherently limits the model’s predictive capacity and causal inference. We will conduct further cohort studies and use follow-up data to further test and optimize the performance of our model.

In summary, the findings from this cohort study of American and European adults suggest that healthy dietary habits may be associated with a lower risk of accelerated aging. However, further studies on the effects of dietary habits on accelerated aging are needed to determine the long-term effects of dietary habits.

## Data Availability

The data used in this study are available from the National Center for Health Statistics of the Centers for Disease Control and Prevention [49].

## Appendix

Multimedia Appendix 1 Subgroup analyses and details on model development for this study.

## Abbreviations

AHEI: Alternative Healthy Eating Index
aMED: Alternative Mediterranean Diet
AUPRC: area under the precision-recall curve
AUROC: area under the receiver operating characteristic curve
DASH: Dietary Approaches to Stop Hypertension
DII: Dietary Inflammatory Index
GBTD: gradient boosting decision tree
HEI-2020: Healthy Eating Index-2020
IHM: Institute of Health and Medicine
KDM: Klemera-Doubal method
MET: metabolic equivalent task
ML: machine learning
NHANES: National Health and Nutrition Examination Survey
PA: physical activity
PIR: poverty income ratio
SHAP: Shapley additive explanations
STROBE: Strengthening the Reporting of Observational Studies in Epidemiology
