# Supplementary material for: Associations Between Dietary Habits and Accelerated Aging and the Establishment of an Accelerated Aging Interpretable Risk Prediction Model via Shapley Additive Explanations: Cross-Sectional Study From Two Representative Populations
Source: JMIR Aging. 2025 Dec 1;8:e72020. doi: 10.2196/72020 (PMC12706446; doi:10.2196/72020)
Supplement: Multimedia Appendix 1 [file aging_v8i1e72020_app1.docx]

**Supplementary Appendix**

[**Supplementary method.** 11](#_Toc209736035)

[**Supplementary Figure 1.** Histogram of dietary index scores 13](#_Toc209736036)

[**Supplementary Figure 2**. Weighted Pearson correlation coefficient between five common dietary indices 14](#_Toc209736037)

[**Supplementary Figure 3.** Associations between dietary index scores and accelerate aging, stratified by BMI in NHANES 15](#_Toc209736038)

[**Supplementary Figure 4.** Associations between dietary index scores and accelerate aging, stratified by BMI in UK Biobank 16](#_Toc209736039)

[**Supplementary Figure 5.** Associations between dietary index scores and accelerate aging, stratified by history of hypertension in NHANES .. 17](#_Toc209736040)

[**Supplementary Figure 6.** Associations between dietary index scores and accelerate aging, stratified by history of hypertension in UK Biobank . 18](#_Toc209736041)

[**Supplementary Figure 7.** Associations between dietary index scores and accelerate aging, stratified by education level in NHANES . 19](#_Toc209736042)

[**Supplementary Figure 8.** Associations between dietary index scores and accelerate aging, stratified by education level in UK Biobank 20](#_Toc209736043)

[**Supplementary Figure 9.** Associations between dietary index scores and accelerate aging, stratified by smoke status in NHANES 21](#_Toc209736044)

[**Supplementary Figure 10.** Associations between dietary index scores and accelerate aging, stratified by smoke status in UK Biobank 22](#_Toc209736045)

[**Supplementary Figure 11**. Restricted cubic spline (RCS) curve of the dietary indices in accelerated aging calculated by KDM Age 23](#_Toc209736046)

[**Supplementary Figure 12.** Restricted cubic spline (RCS) curve of the dietary indices in accelerated aging calculated by PhenoAge. 24](#_Toc209736047)

[**Supplementary Figure 13.** The diagnostic performance of the model in the training set and the test set 25](#_Toc209736048)

[**Supplementary Figure 14.** The precision-recall (PR) performance of the model in the training set and the test set 26](#_Toc209736049)

[**Supplementary Figure 15.** Comparison of the predictive effects of different dietary habit indices on accelerated aging 27](#_Toc209736050)

[**Supplementary Figure 16.** SHAP decision plot for interpreting individual’s prediction outcomes. 28](#_Toc209736051)

[**Supplementary Table 1.** Demographic characteristics of participants in UK Biobank 29](#_Toc209736052)

[**Supplementary Table 2.** Linear regression analysis in UK Biobank 32](#_Toc209736053)

[**Supplementary Table 3.** Quartile regression and linear trend analysis in UK Biobank 33](#_Toc209736054)

[**Supplementary Table 4.** Comparison of discrimination characteristics among different models 35](#_Toc209736055)

[**Supplementary Table 5.** Hyperparameter values of models 36](#_Toc209736056)

[**Supplementary reference** 38](#_Toc209736057)

**Supplementary method.** Detailed description and calculation of healthy diet indices

**1. AHEI**

The AHEI aims to assess diet quality and healthiness, based on nutritional factors related to cardiovascular health and longevity ^[1, 2]^. AHEI, created in 2002, originates from 11 food components and scores, with a scoring range of 0-110. Higher AHEI scores are closely associated with reduced risks of severe chronic diseases and lower cardiovascular mortality. As an indicator of adherence to high-quality dietary guidelines, this score emphasizes fruits, vegetables, and whole grains, while minimizing sodium intake, red meat, and processed meats. ^[2]^. Trans fats were not calculated in this paper because NHANES did not include them in the nutritional dataset, and since 2005, the concentration of trans fats in food has been changing annually, so the AHEI-2010 index in this paper ranges from 0-100 ^[3]^.

**2. DASH**

The DASH diet emphasizes consuming plenty of fruits, vegetables, and low-fat dairy. ^[4]^. We calculated the DASH score for each participant. For each component, participants were divided into five categories based on their intake levels. Component scores for fruits, vegetables, nuts and legumes, low-fat dairy, and whole grains were assigned based on each individual's ranking within the entire cohort. Participants in the top 20% for intake of fruits and juices, vegetables, nuts and legumes, plant-based proteins, whole grains, and low-fat dairy products were awarded 5 points, whereas those in the bottom 20% received 1 point. Participants in the highest quintile for sodium, red/processed meats, and sugary beverages were awarded 1 point, while those in the lowest quintile received 5 points. The scores for each component were aggregated to calculate a total DASH score, which spanned from 8 to 40. Due to the difficulty of accurately measuring sodium intake via FFQ, quintile-based scoring minimizes the risk of misclassification.

**3. HEI2020**

The HEI2020 assessment is based on the nutrient density of various food intakes (e.g., per 1000 calories) rather than absolute quantities, and relies on a set of universal standards applicable to individuals. The HEI2020 total score reflects the overall diet quality and the scores of its components, collectively revealing the quality patterns across various dietary dimensions ^[5]^. Higher HEI2020 scores signify superior diet quality. HEI2020, an updated version of HEI2015, incorporates the latest dietary guidelines and scientific research which maintains the assessment of various food group intakes, with revisions to some factors to more accurately mirror healthy diet standards ^[6]^.

**4. DII**

The DII is an index used to assess the impact of diet on inflammation levels in the body ^[7]^. It considers the inflammatory potential of various foods and nutrients. The DII is designed to compare the inflammatory potential of individual diets based on 45 pro-inflammatory and anti-inflammatory food parameters. DII scoring ranges from anti-inflammatory (low DII score) to pro-inflammatory (high DII score), characterizing the inflammatory nature of participants' diets ^[8, 9]^.

**5. aMED**

The aMED scoring system was adapted from Trichopoulou et al.'s Mediterranean Diet Scale 8^[10]^. The components of aMED include vegetables (excluding potatoes), fruits, nuts, whole grains, legumes, fish, the ratio of monounsaturated to saturated fats, red and processed meats, and alcohol. Participants with an intake above the median in these categories receive 1 point; otherwise, they receive 0 points. Consumption of red and processed meats below the median earns 1 point. Alcohol intake between 5-15 g per day earns 1 point (roughly equates to 12 ounces of regular beer, 5 ounces of wine, or 1.5 ounces of spirits). The possible scoring range for aMED was 0-9, with higher scores indicating greater adherence to the Mediterranean diet habits.

**
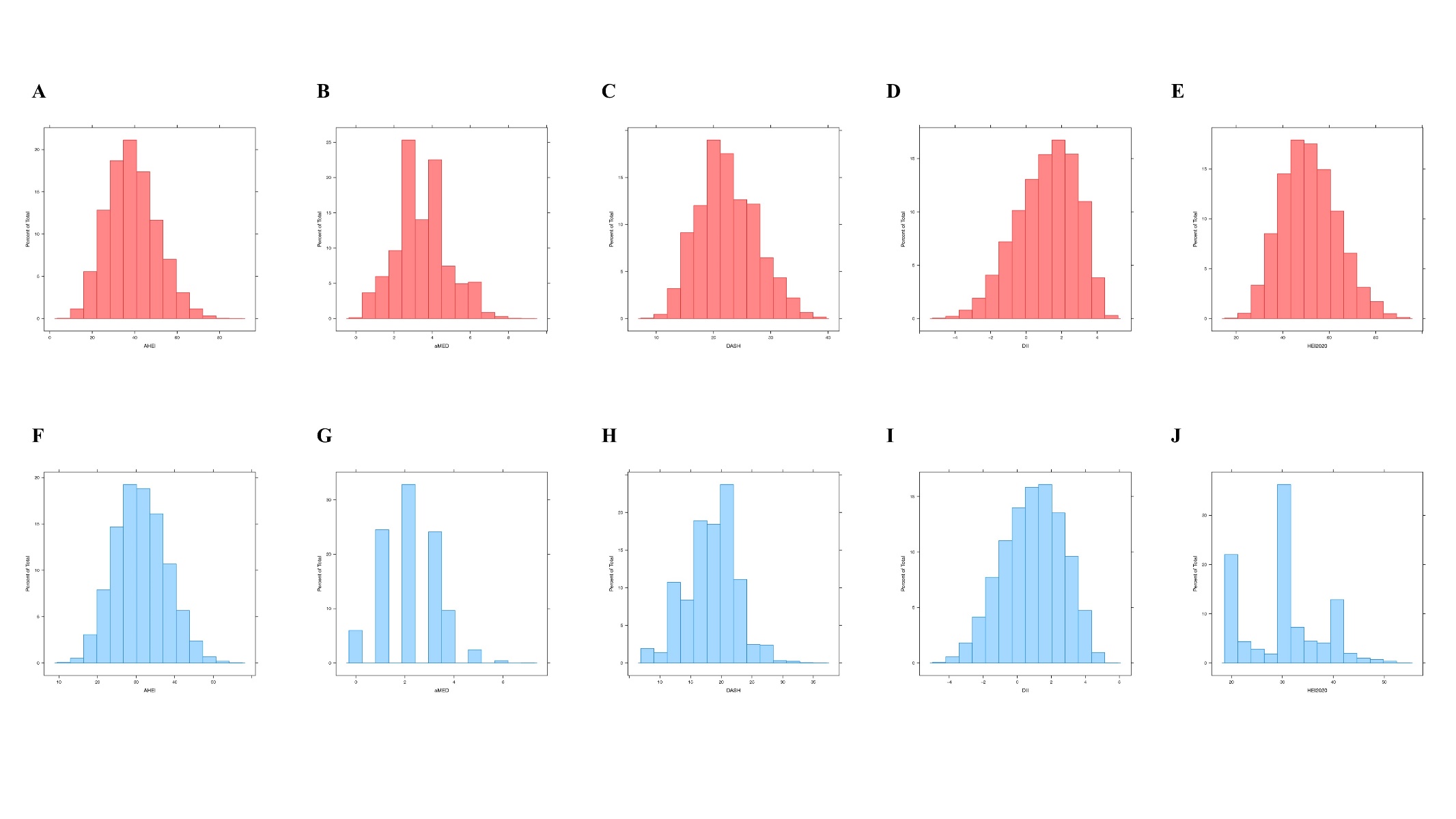
Supplementary Figure 1.** Histogram of dietary index scores. Weighted distribution of AHEI (A), aMED (B), DASH (C), DII (D) and HEI2020 (E) among members of the National Health and Nutrition Examination Survey and distribution of AHEI (F), aMED (G), DASH (H), DII (I) and HEI2020 (J) among members of the UK Biobank.

# **
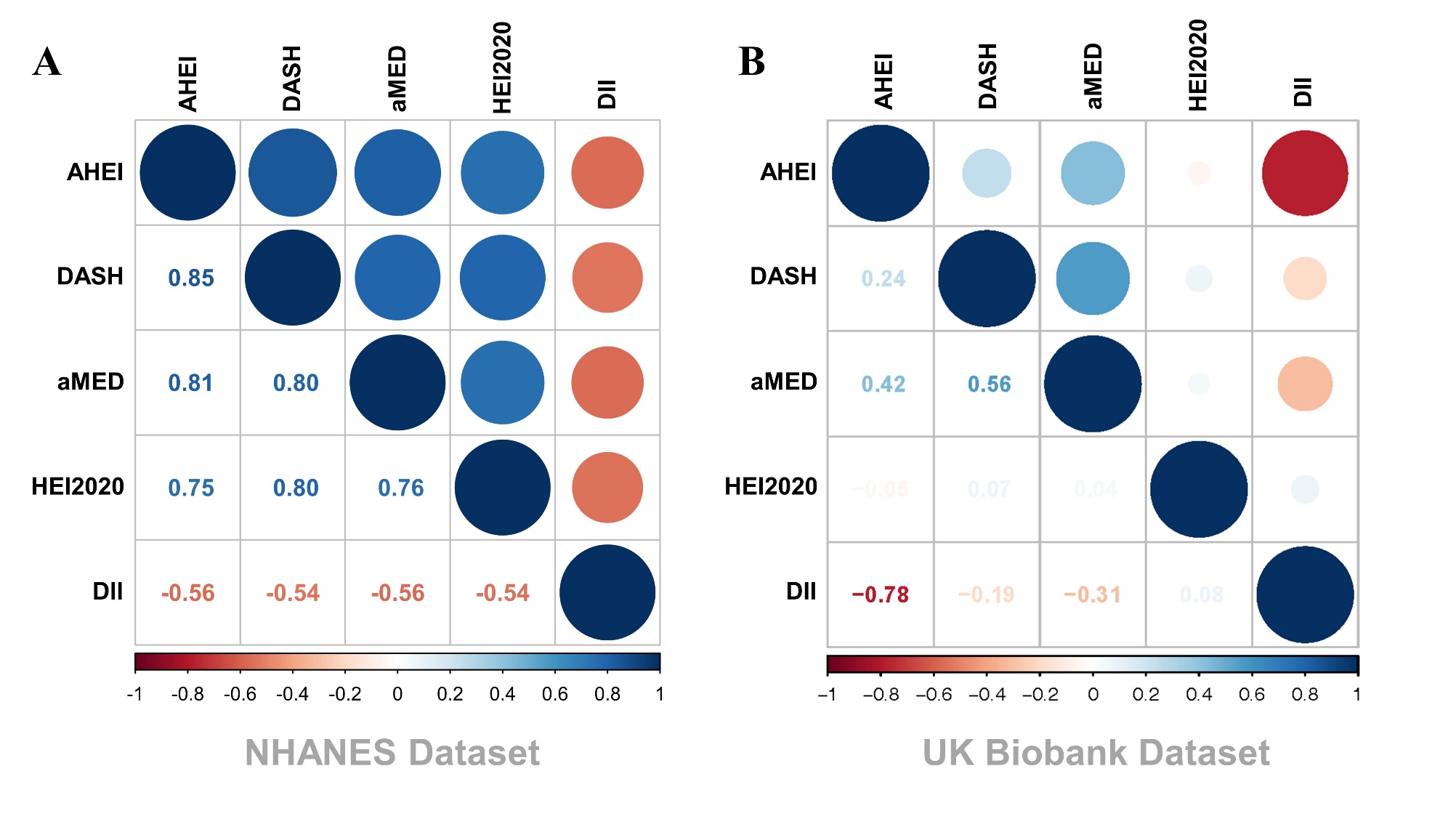
Supplementary Figure 2**. Weighted Pearson correlation coefficient between five common dietary indices in (A) NHANES and (B) UK Biobank. The blue circles indicate positively correlations and the red circle indicate negative correlations.

**
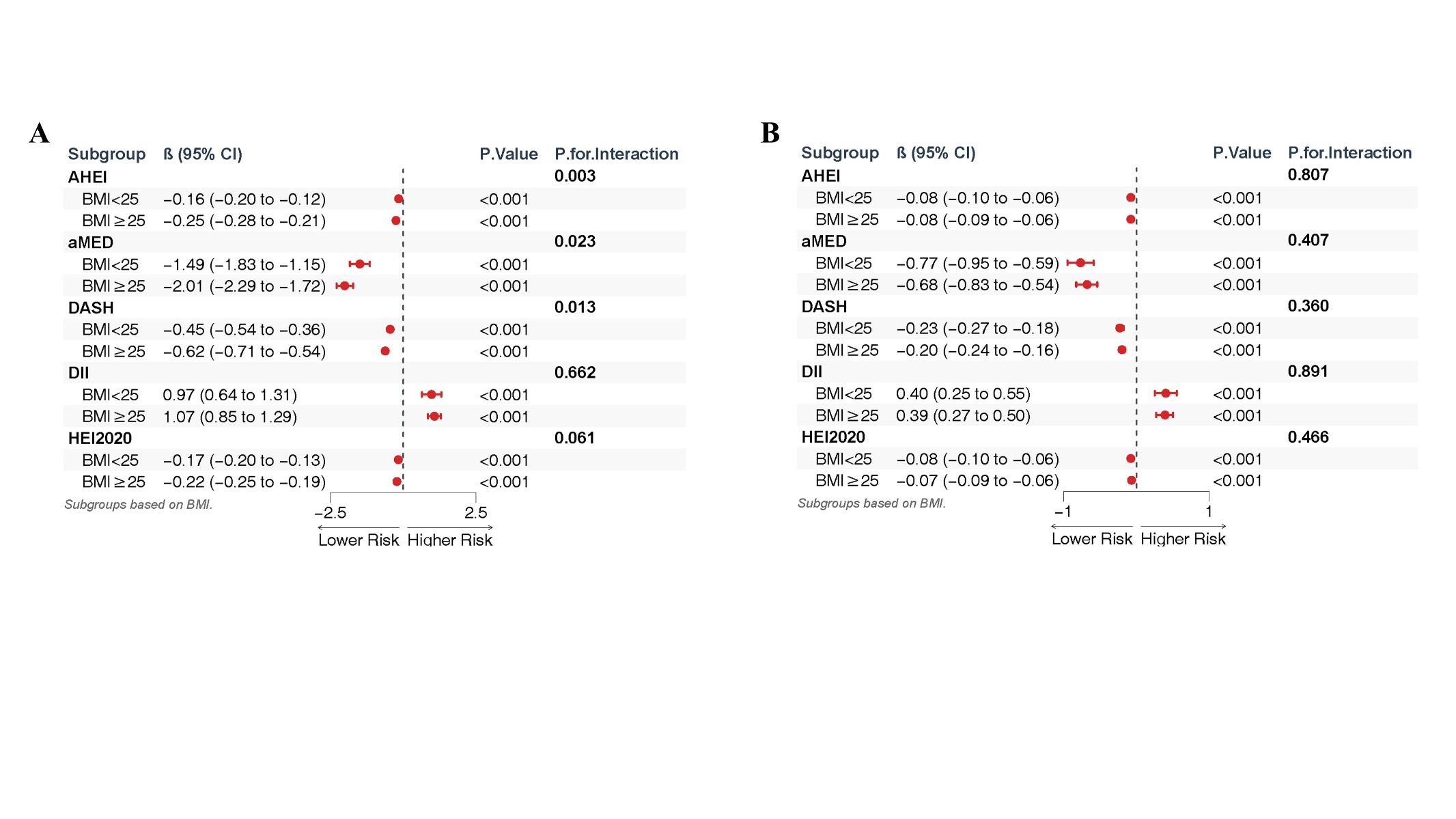
Supplementary Figure 3.** Associations between dietary index scores and accelerate aging, stratified by BMI in NHANES. Biological accelerate aging was calculated using KDM Age (A) and PhenoAge (B). Plots display the β coefficient and 95% confidence interval of the adjusted linear regression model.

**
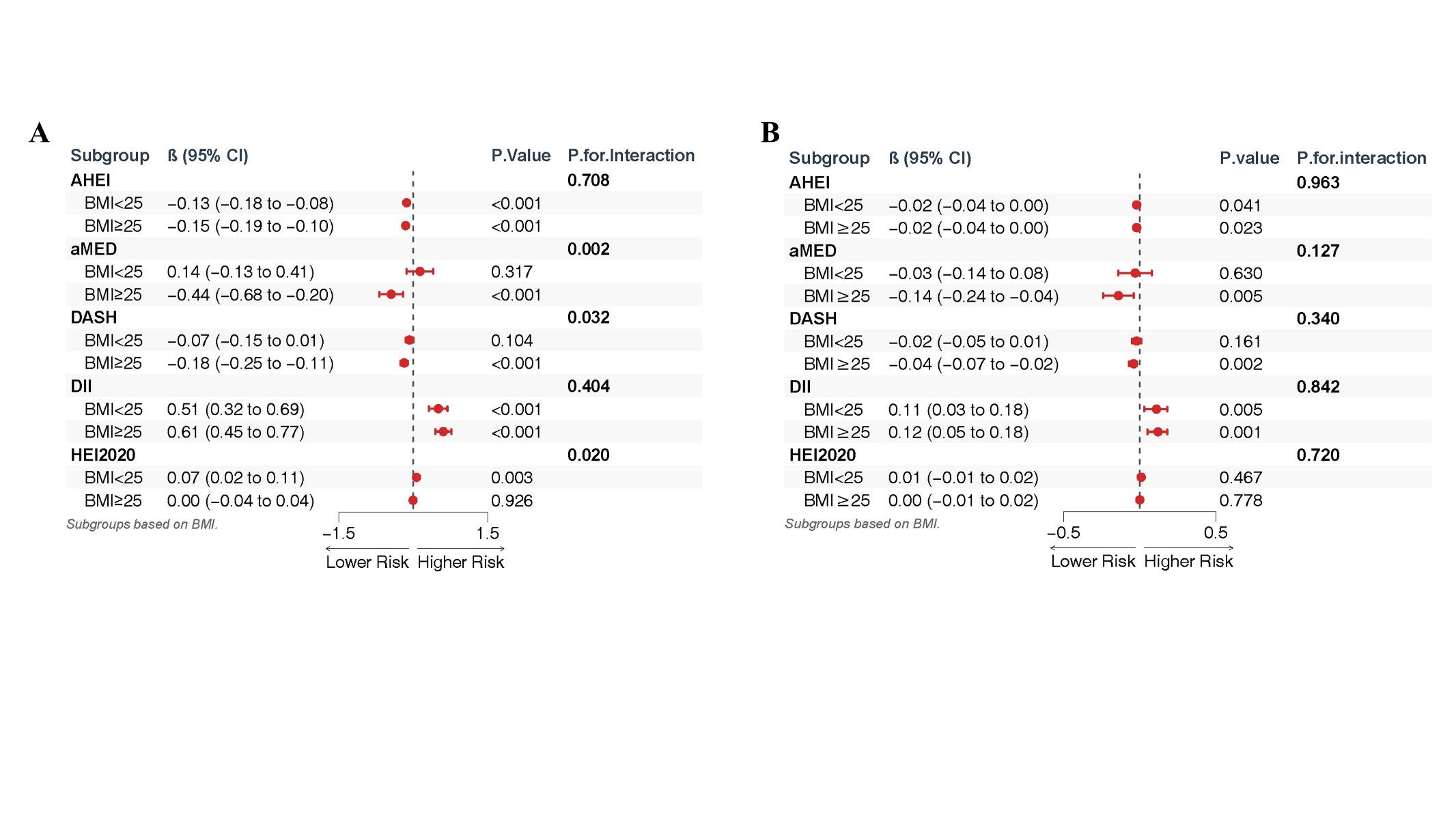
Supplementary Figure 4.** Associations between dietary index scores and accelerate aging, stratified by BMI in UK Biobank. Biological accelerate aging was calculated using KDM Age (A) and PhenoAge (B). Plots display the β coefficient and 95% confidence interval of the adjusted linear regression model.

#
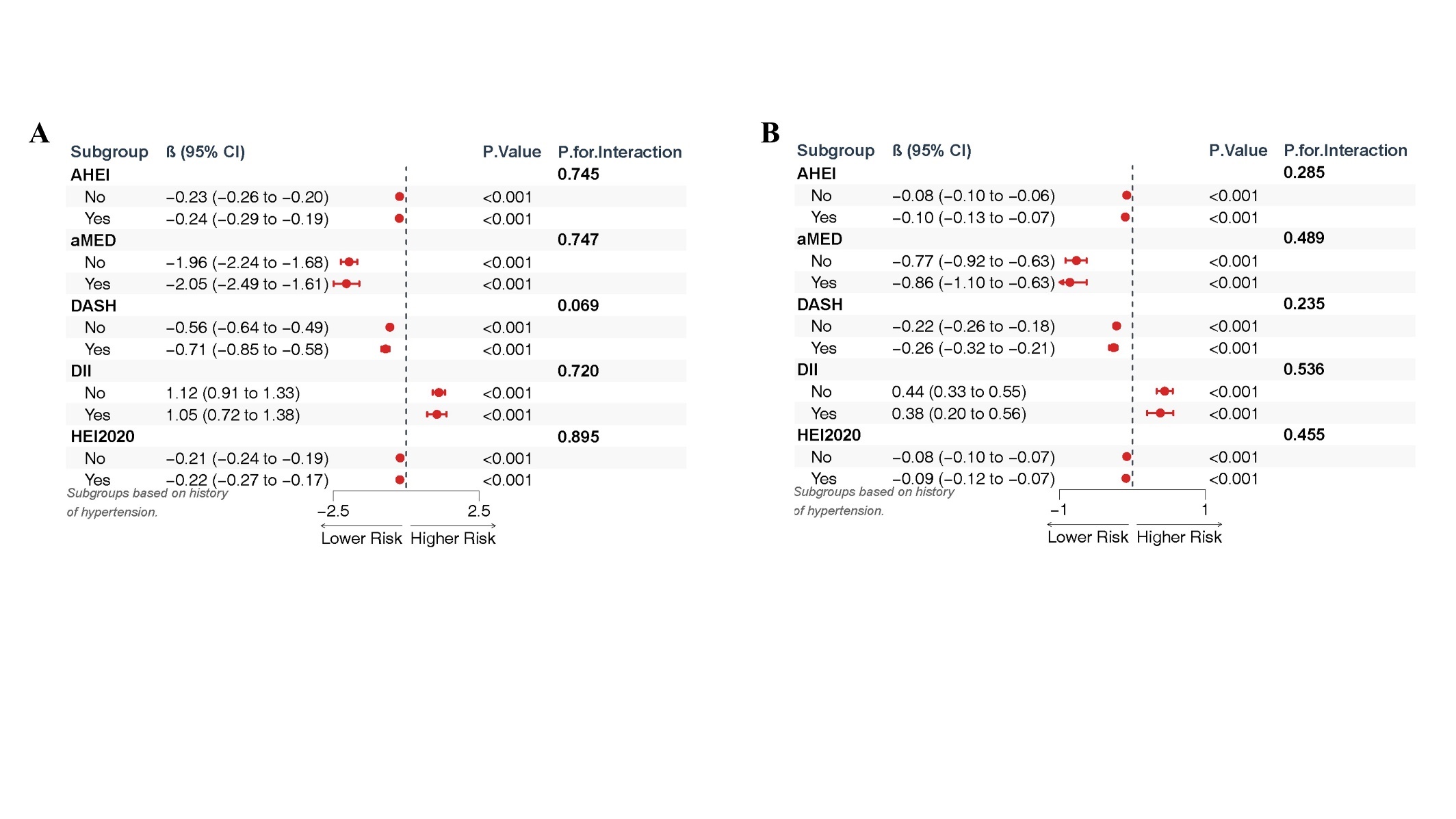
**Supplementary Figure 5.** Associations between dietary index scores and accelerate aging, stratified by history of hypertension in NHANES. Biological accelerate aging was calculated using KDM Age (A) and PhenoAge (B). Plots display the β coefficient and 95% confidence interval of the adjusted linear regression model.

#
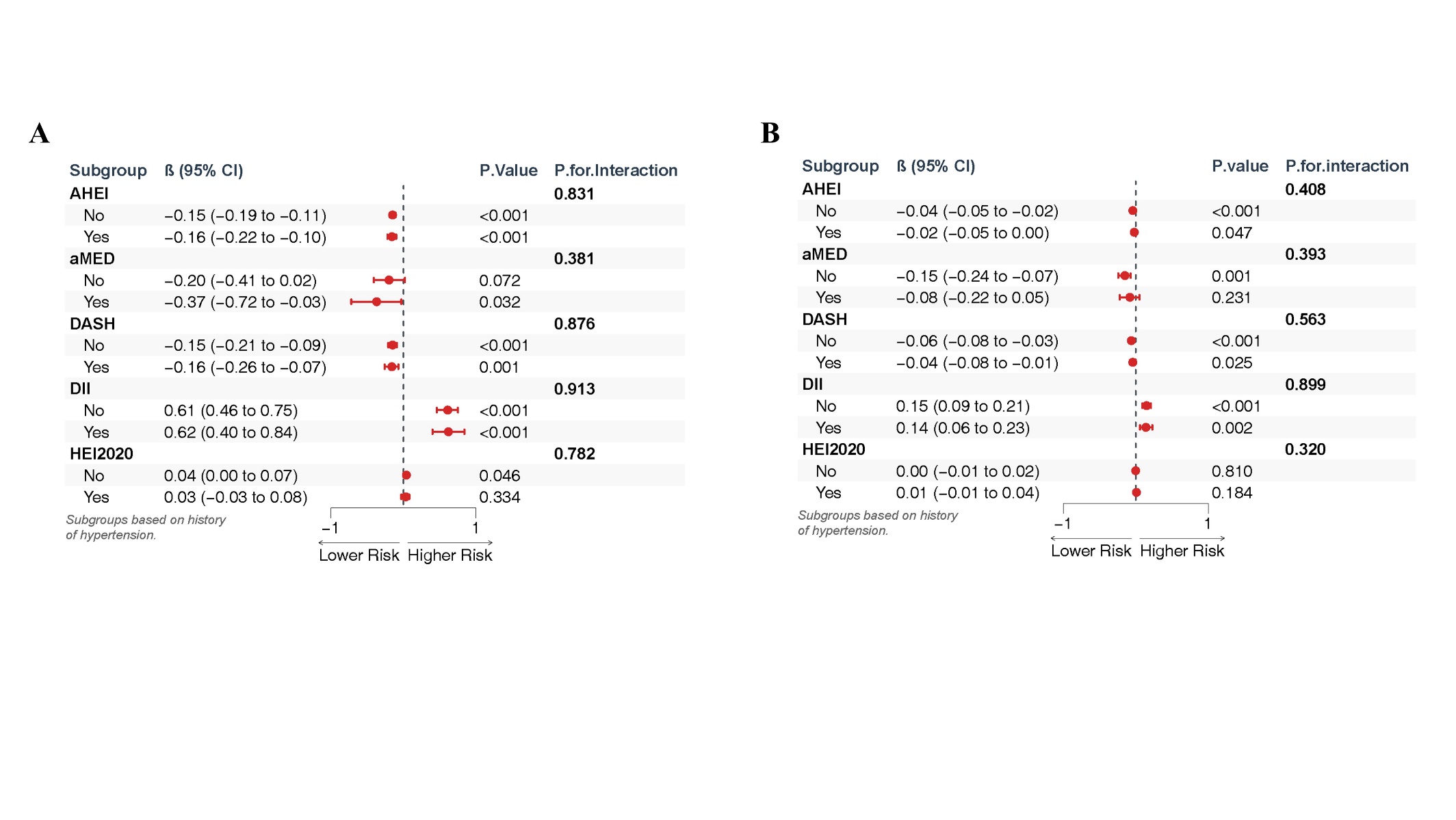
 **Supplementary Figure 6.** Associations between dietary index scores and accelerate aging, stratified by history of hypertension in UK Biobank. Biological accelerate aging was calculated using KDM Age(A) and PhenoAge (B). Plots display the β coefficient and 95% confidence interval of the adjusted linear regression model.


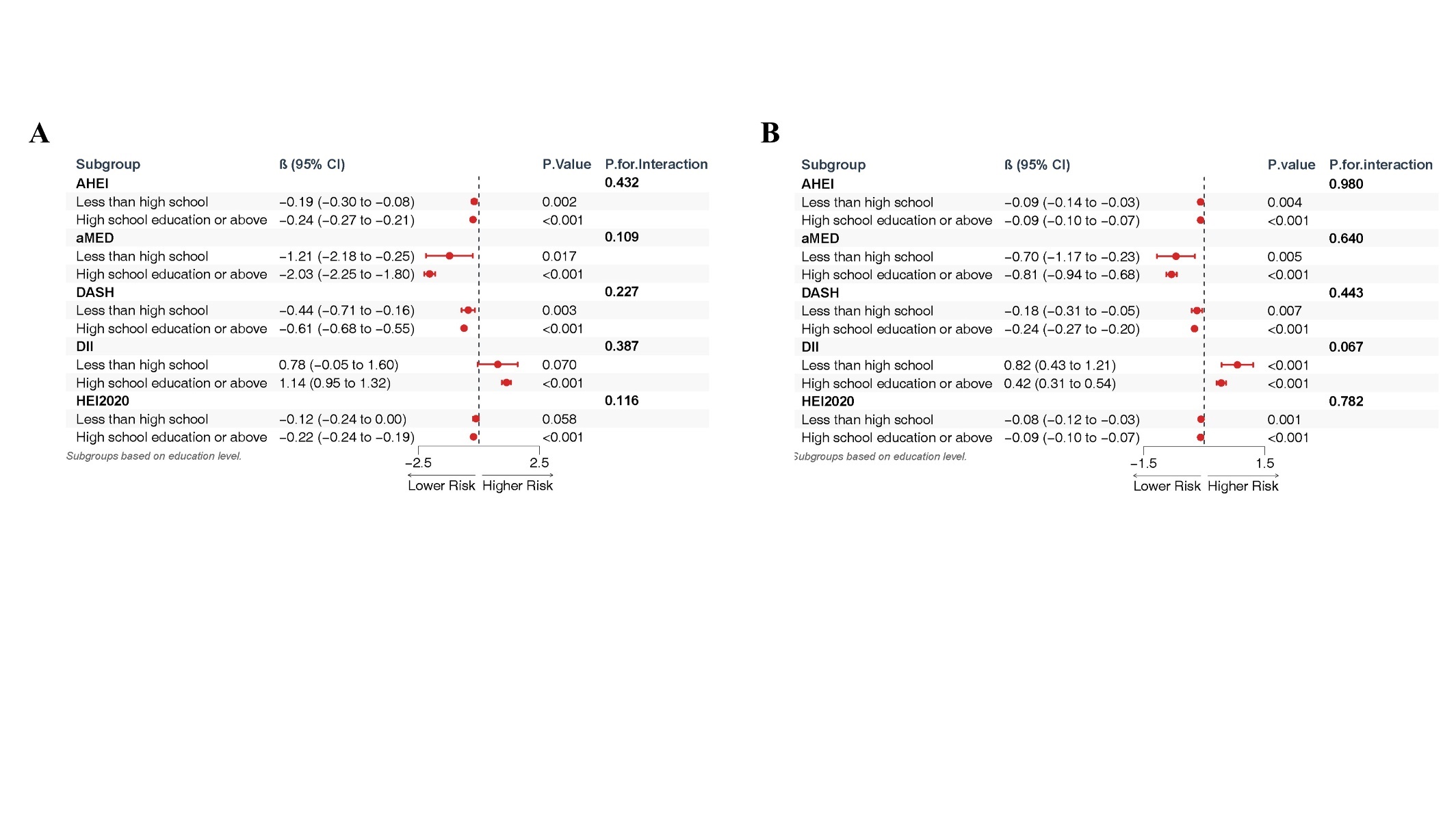


**Supplementary Figure 7.** Associations between dietary index scores and accelerate aging, stratified by education level in NHANES. Biological accelerate aging was calculated using KDM Age(A) and PhenoAge (B). Plots display the β coefficient and 95% confidence interval of the adjusted linear regression model.


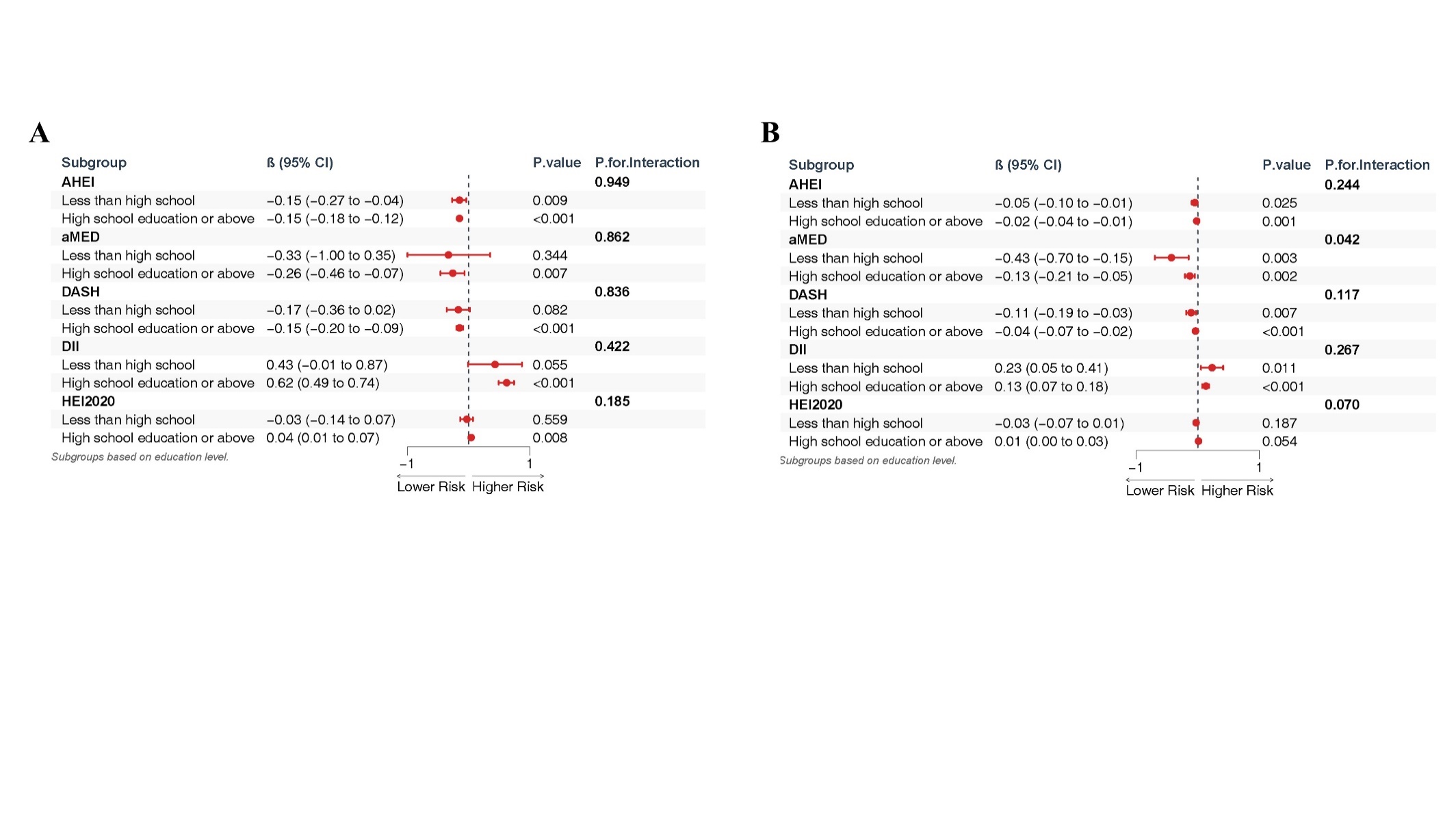


**Supplementary Figure 8.** Associations between dietary index scores and accelerate aging, stratified by education level in UK Biobank. Biological accelerate aging was calculated using KDM Age (A) and PhenoAge (B). Plots display the β coefficient and 95% confidence interval of the adjusted linear regression model.

#
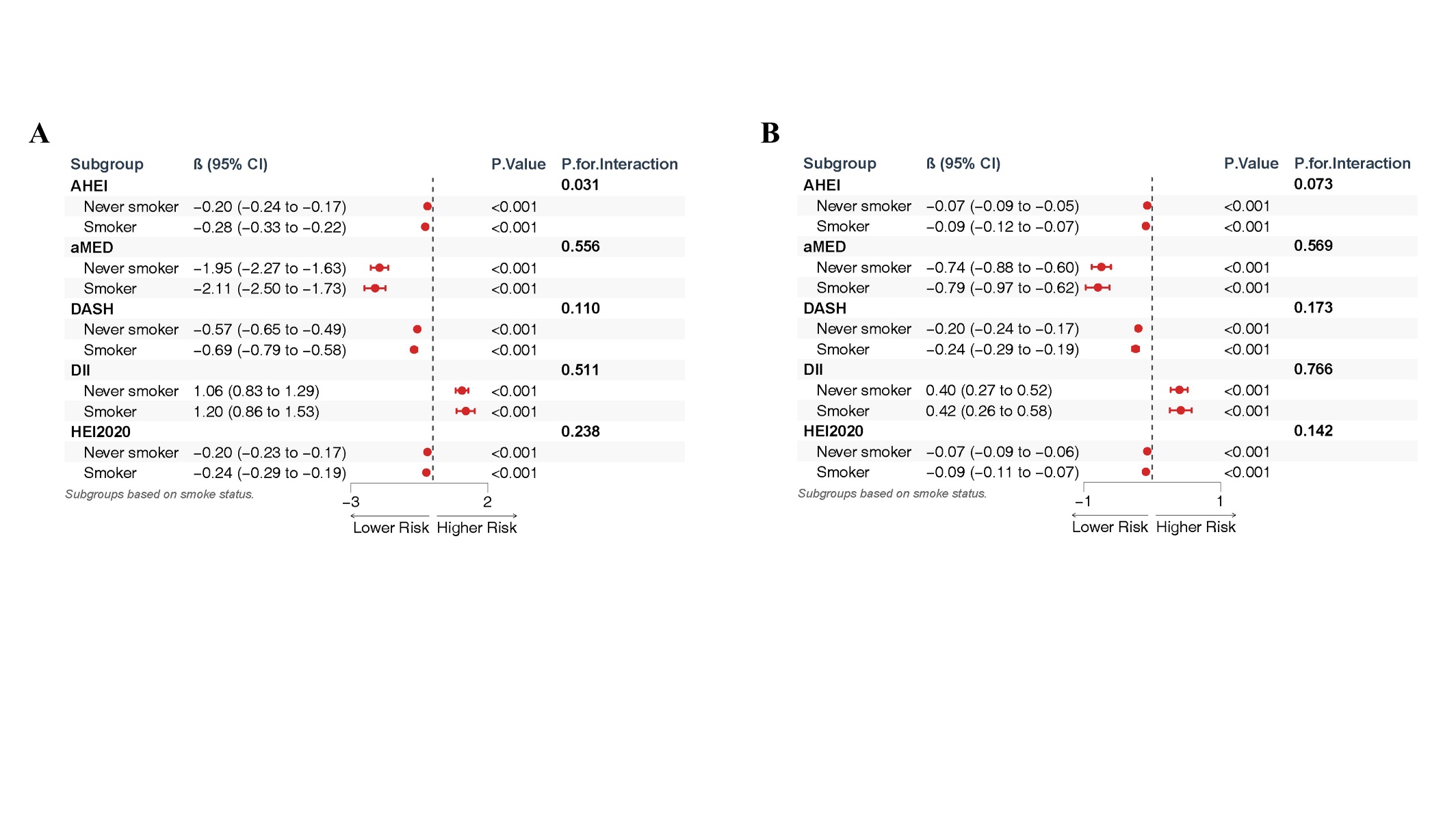
**Supplementary Figure 9.** Associations between dietary index scores and accelerate aging, stratified by smoke status in NHANES. Biological accelerate aging was calculated using KDM Age (A) and PhenoAge (B). Plots display the β coefficient and 95% confidence interval of the adjusted linear regression model.


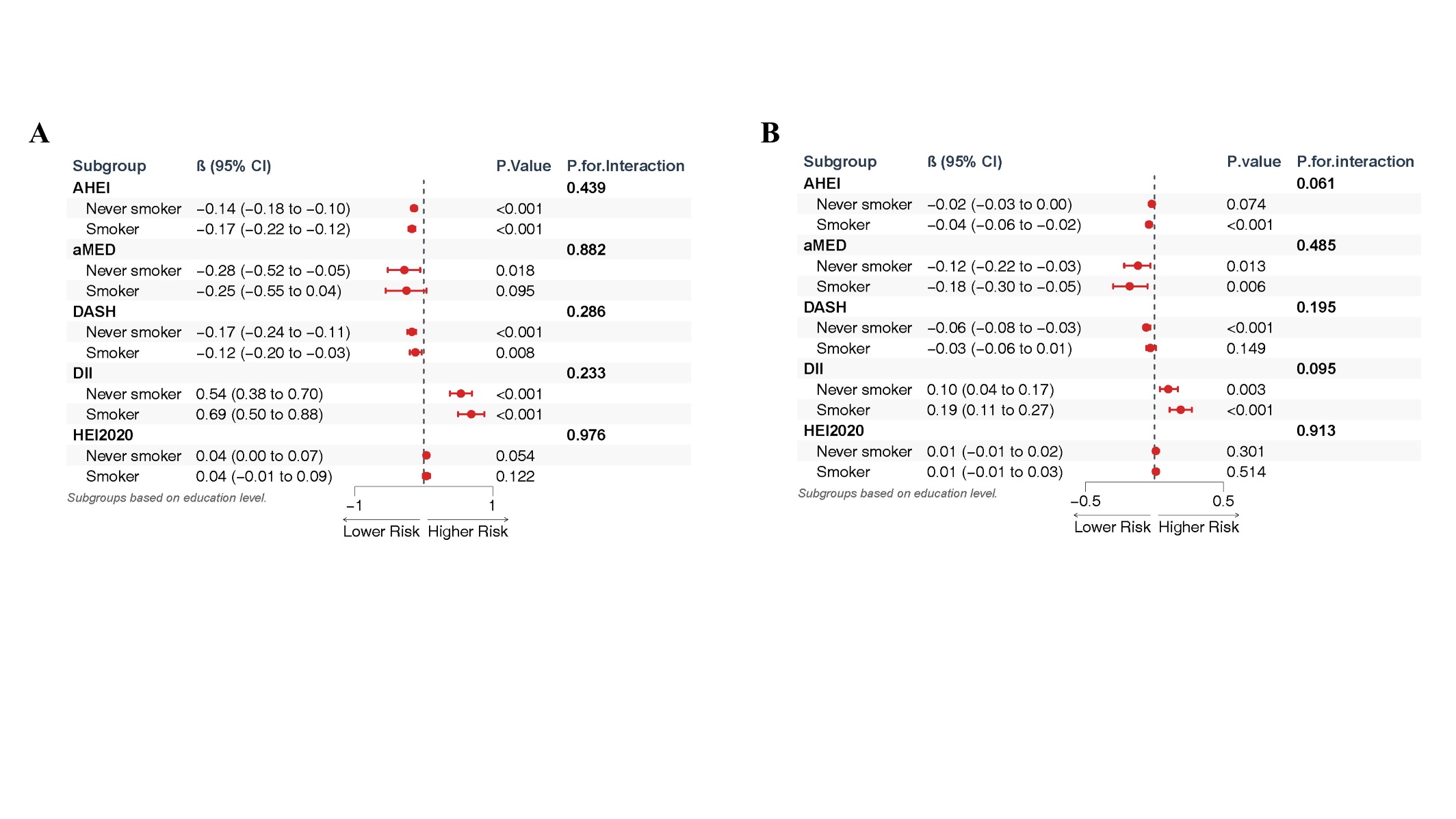


**Supplementary Figure 10.** Associations between dietary index scores and accelerate aging, stratified by smoke status in UK Biobank. Biological accelerate aging was calculated using KDM Age (A) and PhenoAge (B). Plots display the β coefficient and 95% confidence interval of the adjusted linear regression model.

# **
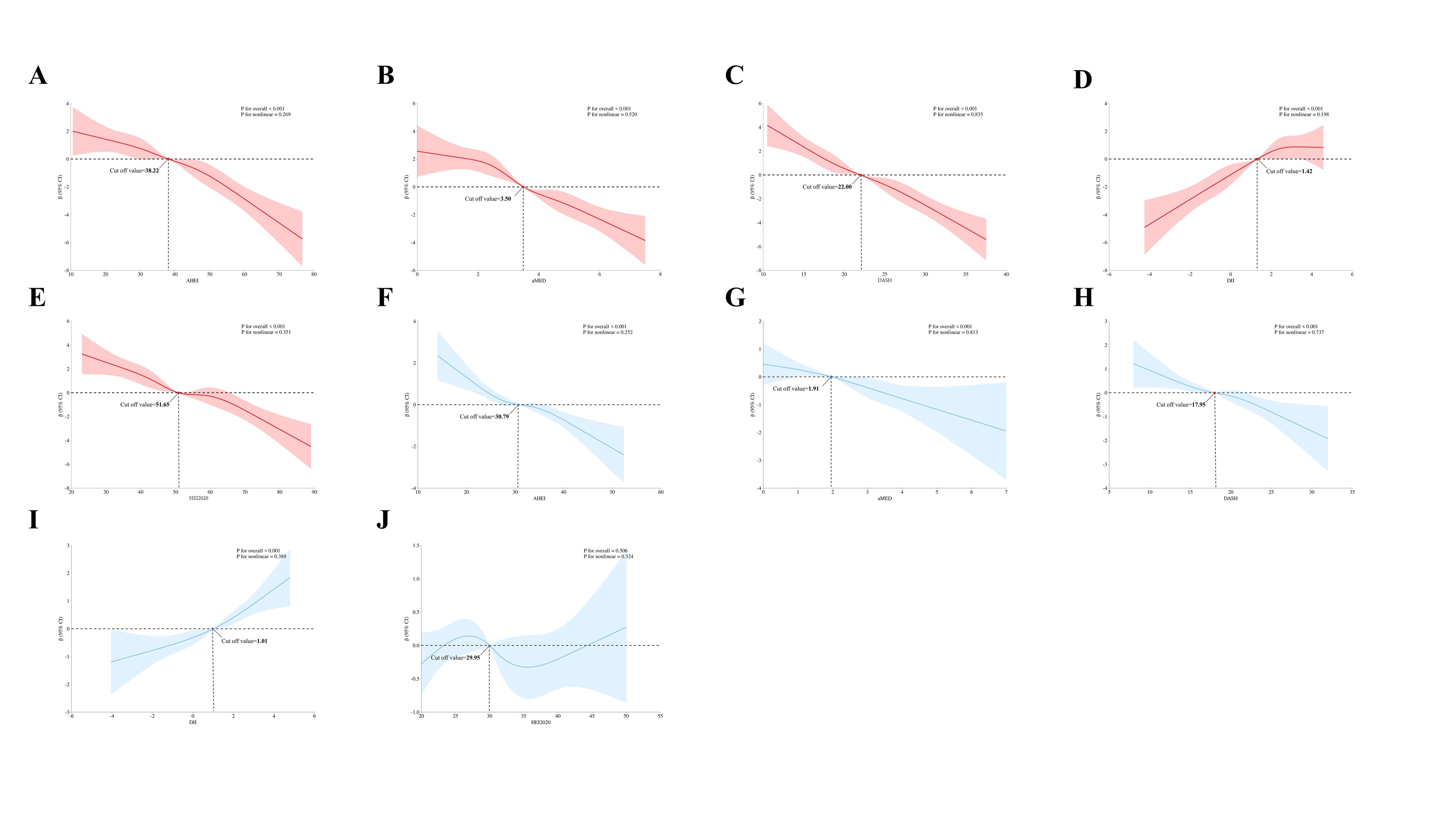
Supplementary Figure 11**. Restricted cubic spline (RCS) curve of the dietary indices in accelerated aging calculated by KDM Age. RCS curve for AHEI in NHANES (A); RCS curve for aMED in NHANES (B); RCS curve for DASH in NHANES (C); RCS curve for DII in NHANES (D); RCS curve for HEI2020 in NHANES (E); RCS curve for AHEI in UK Biobank (F); RCS curve for aMED in UK Biobank (G); RCS curve for DASH in UK Biobank (H); RCS curve for DII in UK Biobank (I); RCS curve for HEI2020 in UK Biobank (J). Lines represent β values, and area shaded represents the 95% confidence interval (CI).

#
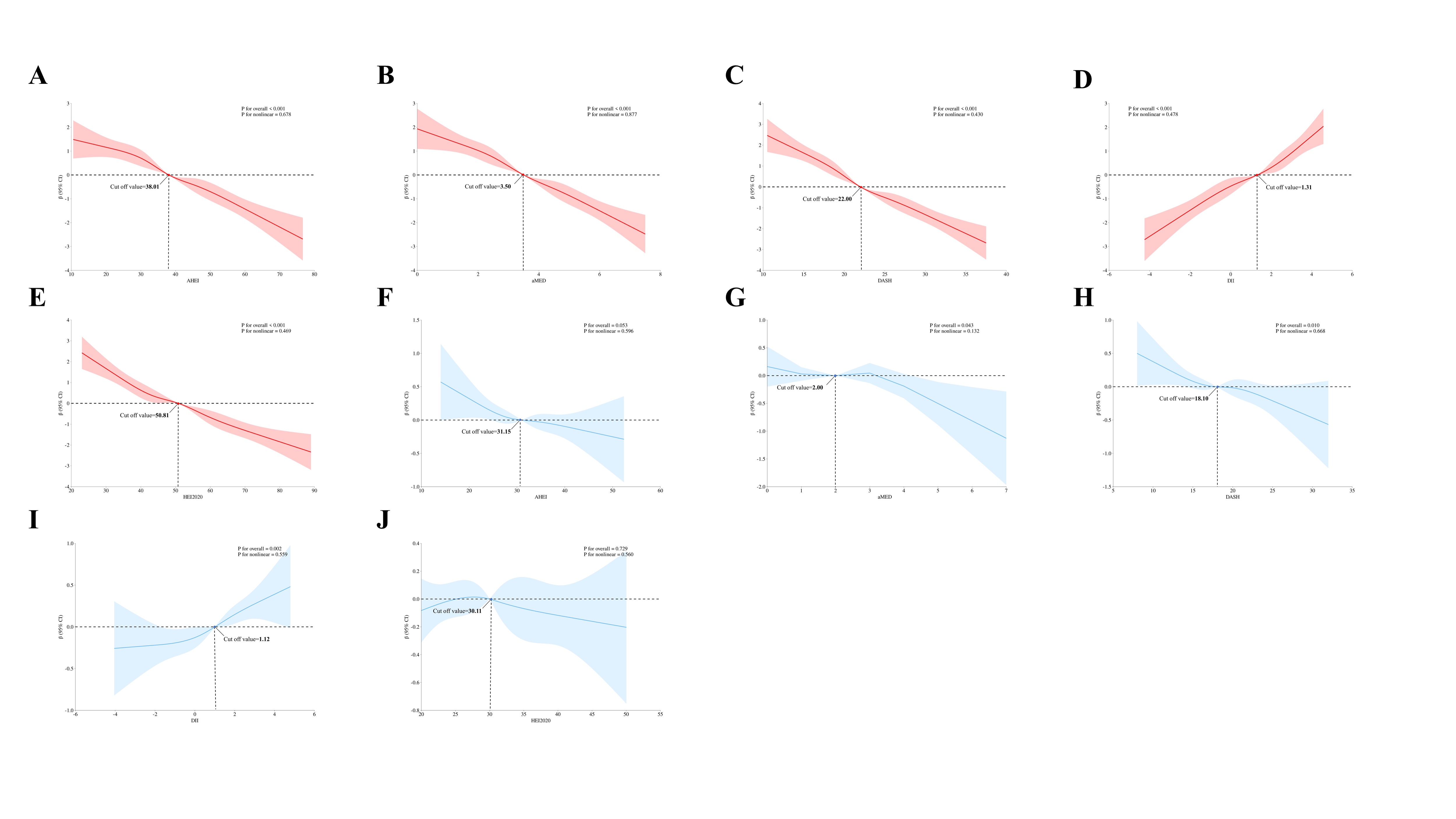
**Supplementary Figure 12.** Restricted cubic spline (RCS) curve of the dietary indices in accelerated aging calculated by PhenoAge. RCS curve for AHEI in NHANES (A); RCS curve for aMED in NHANES (B); RCS curve for DASH in NHANES (C); RCS curve for DII in NHANES (D); RCS curve for HEI2020 in NHANES (E); RCS curve for AHEI in UK Biobank (F); RCS curve for aMED in UK Biobank (G); RCS curve for DASH in UK Biobank (H); RCS curve for DII in UK Biobank (I); RCS curve for HEI2020 in UK Biobank (J). Lines represent β values, and area shaded represents the 95% confidence interval (CI).


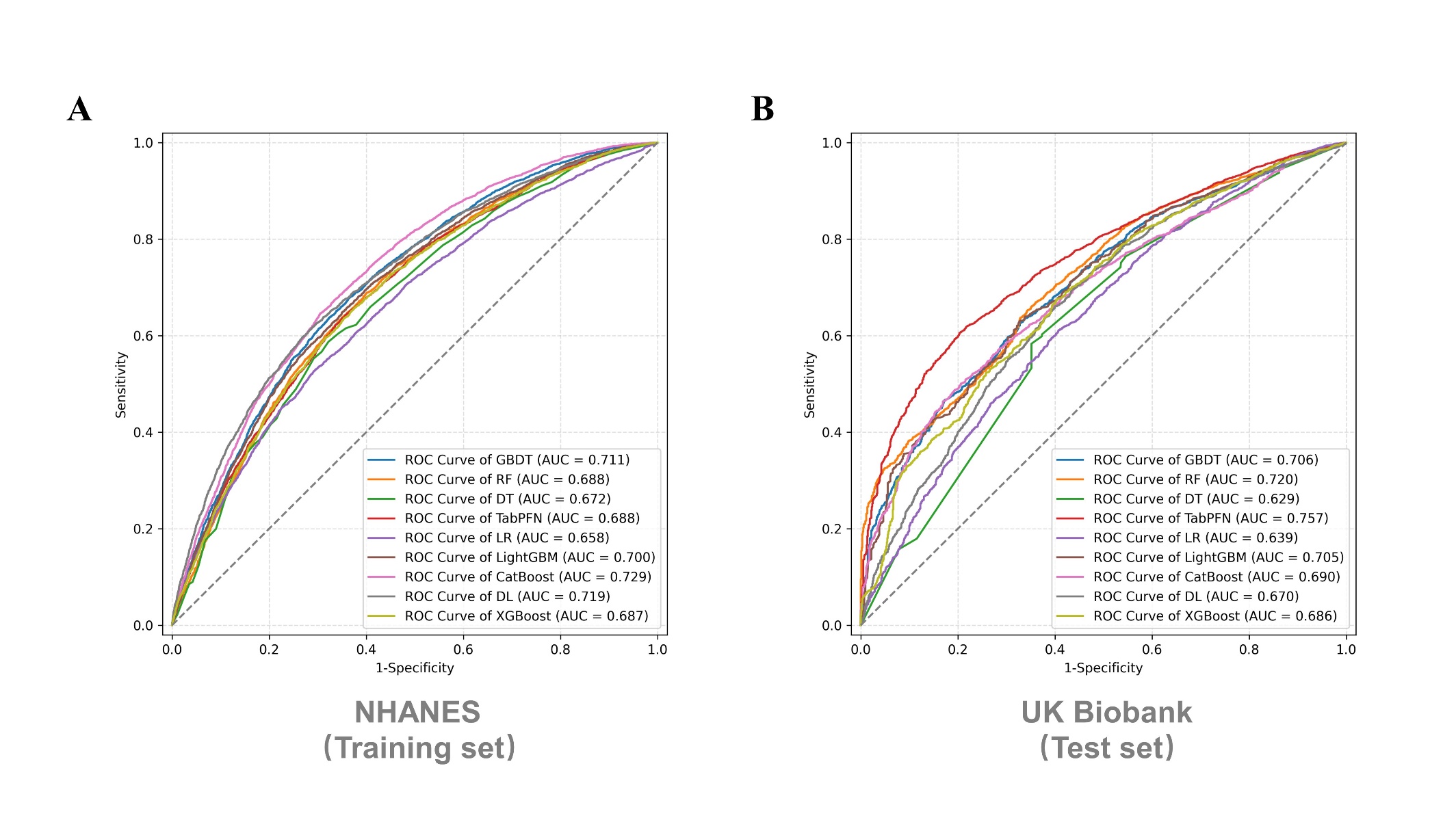


**Supplementary Figure 13.** The diagnostic performance of the model in the training set and the test set. (A) The ROC curve of the model among NHANES participants; (B) The ROC curve of the model among UK Biobank participants


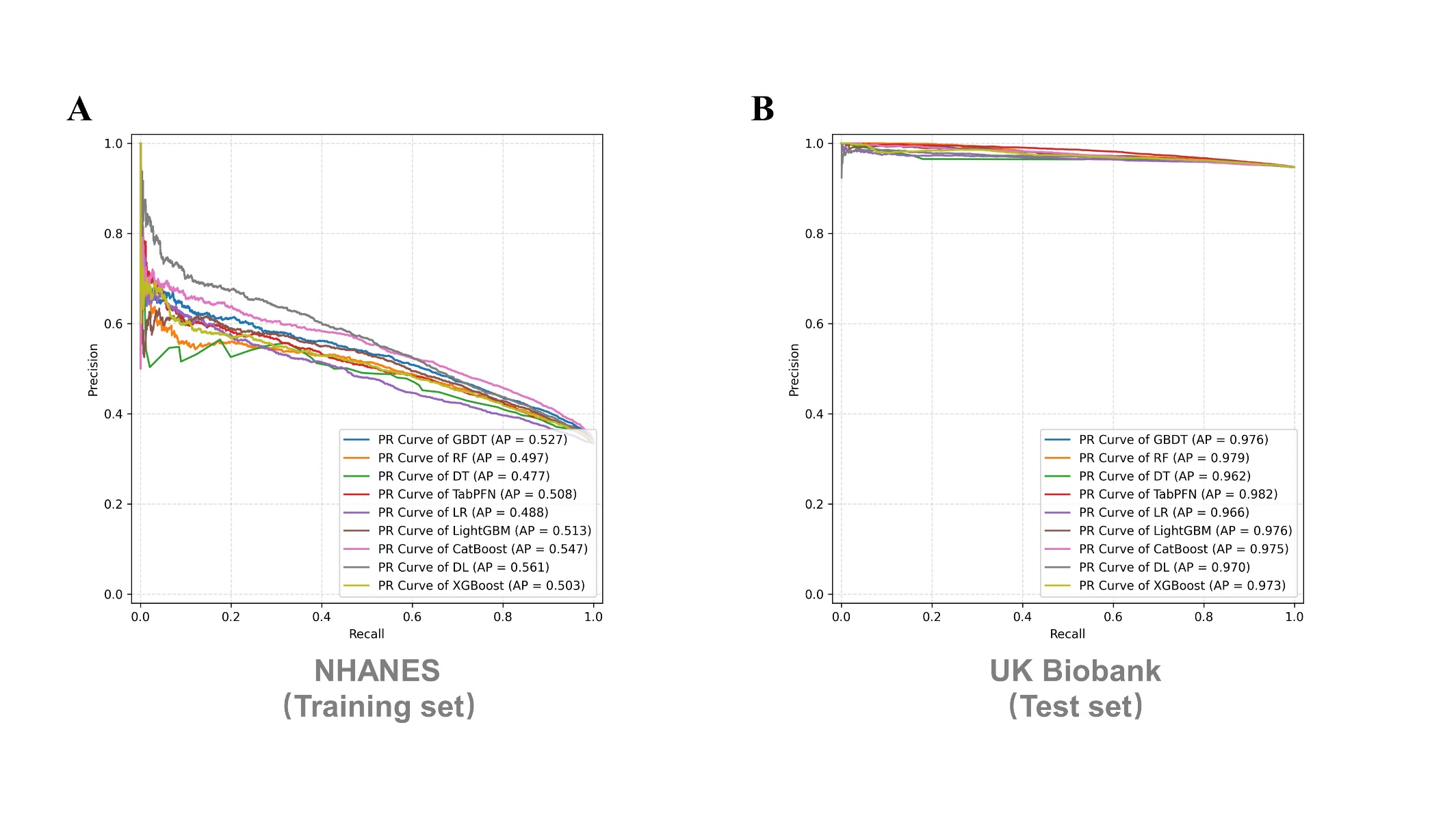


**Supplementary Figure 14.** The precision-recall (PR) performance of the model in the training set and the test set. (A) The PR curve of the model among NHANES participants; (B) The PR curve of the model among UK Biobank participants


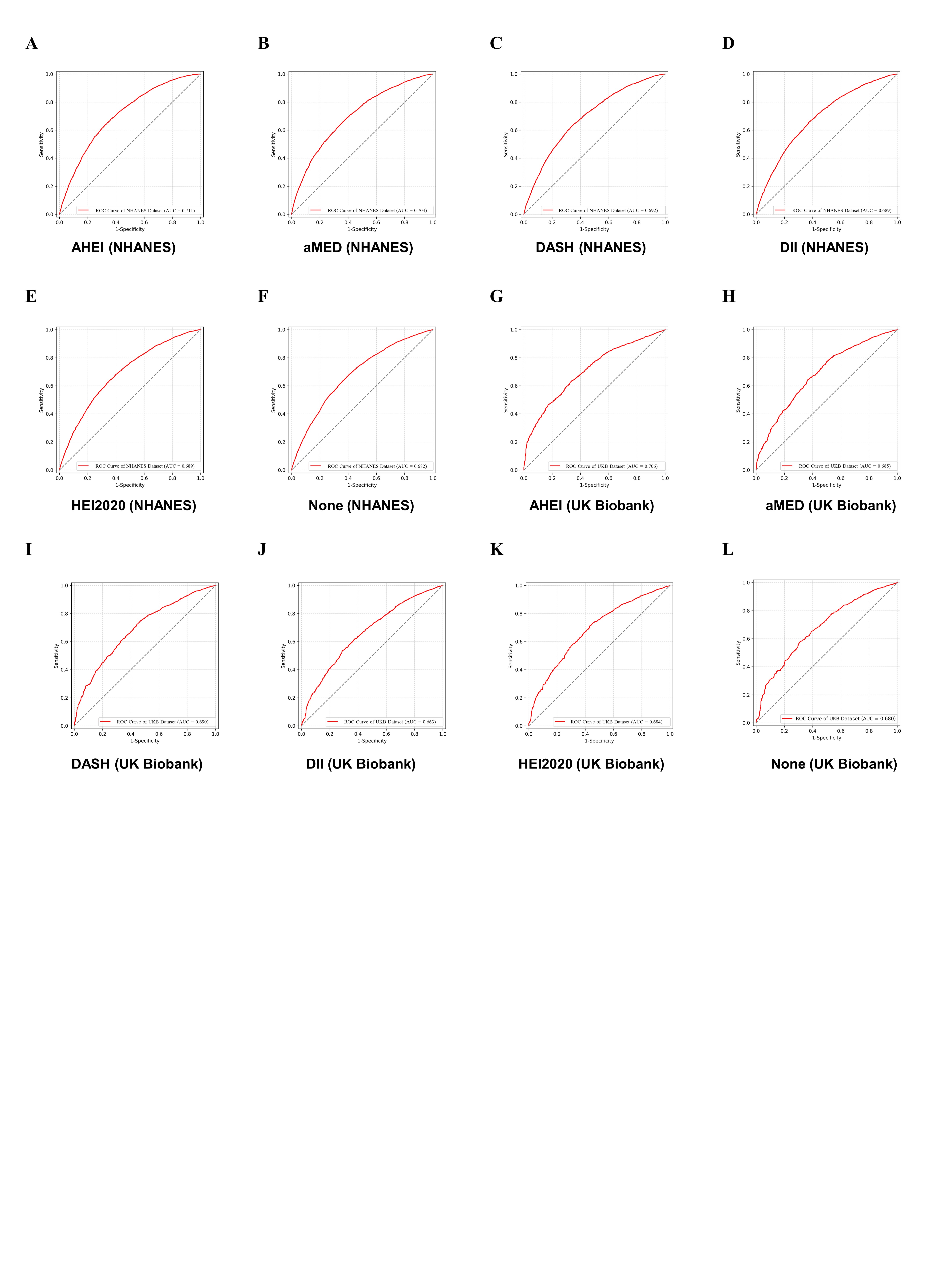


**Supplementary Figure 15.** Comparison of the predictive effects of different dietary habit indices on accelerated aging.


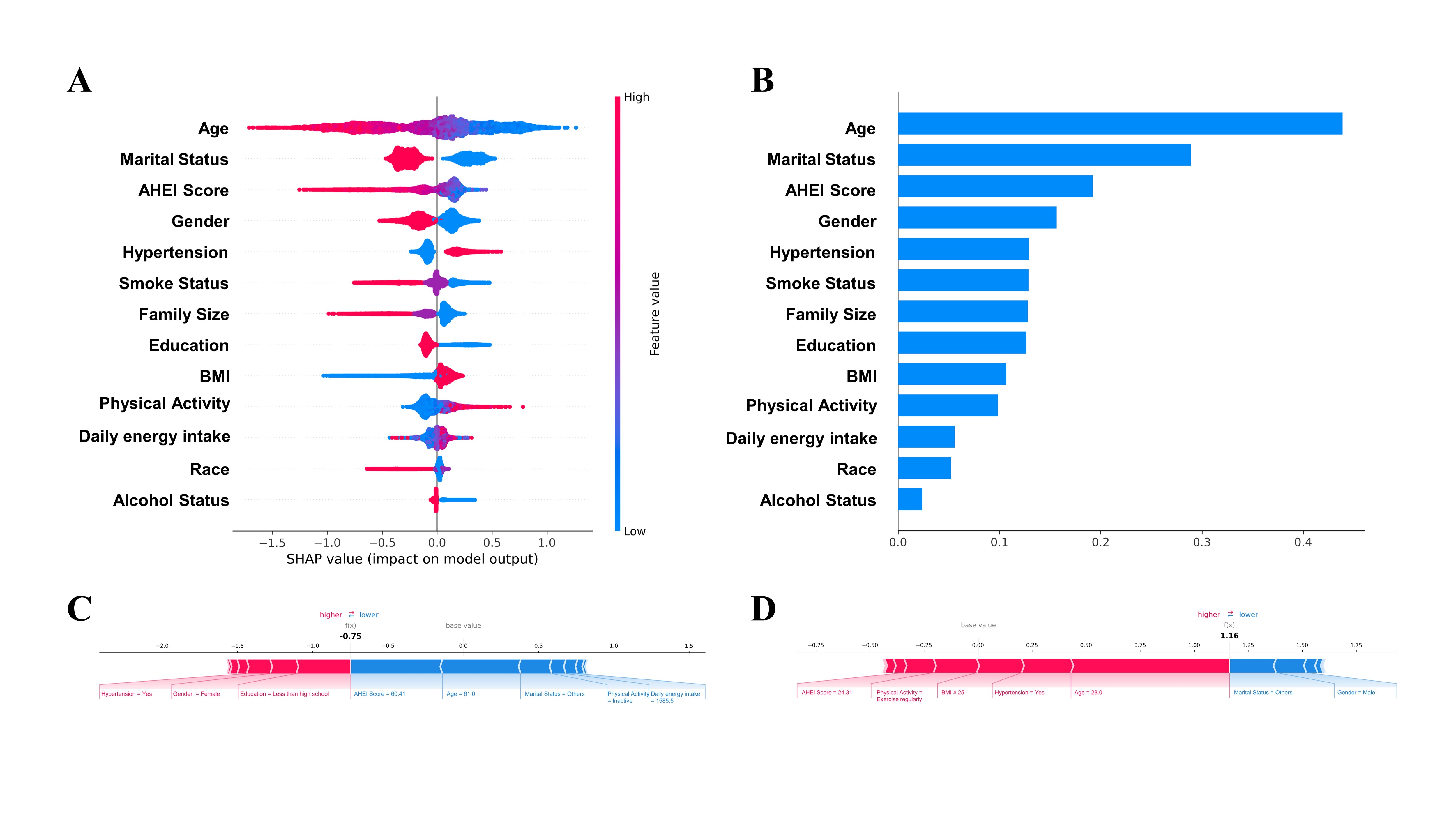


**Supplementary Figure 16.** SHAP decision plot for interpreting individual’s prediction outcomes. (A) SHAP honeycomb diagram of the prediction model. Each point represents a feature value, and different colors represent the final impact of the feature on the model's output results. (B) Ranking of the importance of model prediction features. The abscissa represents the SHAP value, and a larger SHAP value indicates that the variable is more important. (C) SHAP force plot for selected non-accelerated aging participant. (D) SHAP force plot for selected accelerated aging participant.

# **Supplementary Table 1.** Demographic characteristics of participants in UK Biobank

| **Characteristic** | **Overall**, N = 8594 (100%)*^1^* | **KDM Age** | | ***P Value****^2^* | **PhenoAge** | | ***P Value****^2^* |
| --- | --- | --- | --- | --- | --- | --- | --- |
|  |  | **Accelerated aging,** N = 8078 (94%)*^1^* | **Non-accelerated aging,** N = 516 (6%)*^1^* |  | **Accelerated aging,** N = 852 (10%)*^1^* | **Non-accelerated aging,** N = 3646 (90%)*^1^* |  |
| **Age (years)** | 55.98±7.60 | 55.73±7.61 | 59.93±6.28 | <0.001 | 56.08±7.79 | 55.97±7.58 | 0.587 |
| **Sex** |  |  |  | <0.001 |  |  | 0.851 |
| Female | 4,689(54.56%) | 4,688(58.03%) | 1(0.19%) |  | 468(54.93%) | 4,221(54.52%) |  |
| Male | 3,905(45.44%) | 3,390(41.97%) | 515(99.81%) |  | 384(45.07%) | 3,521(45.48%) |  |
| **Race** |  |  |  | 0.028 |  |  | 0.806 |
| Black | 25(0.29%) | 1(0.19%) | 24(0.30%) |  | 22(0.28%) | 3(0.35%) |  |
| White | 8,395(97.68%) | 504(97.67%) | 7,891(97.69%) |  | 7,564(97.70%) | 831(97.54%) |  |
| Other Race | 174(2.02%) | 11(2.13%) | 163(2.02%) |  | 156(2.01%) | 18(2.11%) |  |
| **Education** |  |  |  | 0.003 |  |  | <0.001 |
| High School Grad/GED or Equivalent | 2,361(27.47%) | 2,250(27.85%) | 111(21.51%) |  | 286(33.57%) | 2,075(26.80%) |  |
| Less than high school | 650(7.56%) | 615(7.61%) | 35(6.78%) |  | 76(8.92%) | 574(7.41%) |  |
| More than high school | 5,583(64.96%) | 5,213(64.53%) | 370(71.71%) |  | 490(57.51%) | 5,093(65.78%) |  |
| **Marital Status** |  |  |  | <0.001 |  |  | <0.001 |
| Divorce or living alone | 2,456(28.58%) | 2,348(29.07%) | 108(20.93%) |  | 289(33.92%) | 2,167(27.99%) |  |
| Ohters | 6,138(71.42%) | 5,730(70.93%) | 408(79.07%) |  | 563(66.08%) | 5,575(72.01%) |  |
| **Deprivation Index** |  |  |  | 0.802 |  |  | 0.047 |
| Higher (most deprived) | 2,786(32.42%) | 2,612(32.33%) | 174(33.72%) |  | 304(35.68%) | 2,482(32.06%) |  |
| Lower (least deprived) | 2,938(34.19%) | 2,765(34.23%) | 173(33.53%) |  | 264(30.99%) | 2,674(34.54%) |  |
| Middle | 2,870(33.40%) | 2,701(33.44%) | 169(32.75%) |  | 284(33.33%) | 2,586(33.40%) |  |
| **Family Size** |  |  |  | 0.165 |  |  | <0.001 |
| 1~3 | 6,783(78.96%) | 6,359(78.76%) | 424(82.17%) |  | 714(84.00%) | 6,069(78.41%) |  |
| 4~5 | 1,710(19.91%) | 1,623(20.10%) | 87(16.86%) |  | 131(15.41%) | 1,579(20.40%) |  |
| 6 and more | 97(1.13%) | 92(1.14%) | 5(0.97%) |  | 5(0.59%) | 92(1.19%) |  |
| **Body Mass Index (BMI)** |  |  |  | <0.001 |  |  | <0.001 |
| <20 | 251(2.92%) | 227(2.81%) | 24(4.65%) |  | 9(1.06%) | 242(3.13%) |  |
| >25 | 4,988(58.04%) | 4,786(59.25%) | 202(39.15%) |  | 684(80.28%) | 4,304(55.59%) |  |
| 20-25 | 3,355(39.04%) | 3,065(37.94%) | 290(56.20%) |  | 159(18.66%) | 3,196(41.28%) |  |
| **Hypertension** |  |  |  | 0.002 |  |  | <0.001 |
| No | 5,825(67.78%) | 5,439(67.33%) | 386(74.81%) |  | 385(45.19%) | 5,440(70.27%) |  |
| Yes | 2,769(32.22%) | 2,639(32.67%) | 130(25.19%) |  | 467(54.81%) | 2,302(29.73%) |  |
| **Physical Activity** |  |  |  | 0.025 |  |  | <0.001 |
| Exercise regularly | 4,715(54.86%) | 307(59.50%) | 4,408(54.57%) |  | 4,315(55.73%) | 400(46.95%) |  |
| Inactive | 1,107(12.88%) | 48(9.30%) | 1,059(13.11%) |  | 951(12.28%) | 156(18.31%) |  |
| Moderately active | 2,772(32.26%) | 161(31.20%) | 2,611(32.32%) |  | 2,476(31.98%) | 296(34.74%) |  |
| **Smoking Status** |  |  |  | 0.024 |  |  | <0.001 |
| Current smoker | 324(3.77%) | 314(3.89%) | 10(1.94%) |  | 71(8.33%) | 253(3.27%) |  |
| Ever smoker | 3,075(35.78%) | 2,872(35.55%) | 203(39.34%) |  | 356(41.78%) | 2,719(35.12%) |  |
| Never smoker | 5,195(60.45%) | 4,892(60.56%) | 303(58.72%) |  | 425(49.88%) | 4,770(61.61%) |  |
| **Alcohol Consumption** |  |  |  | <0.001 |  |  | 0.644 |
| No | 4,073(47.39%) | 3,874(47.96%) | 199(38.57%) |  | 397(46.60%) | 3,676(47.48%) |  |
| Yes | 4,521(52.61%) | 4,204(52.04%) | 317(61.43%) |  | 455(53.40%) | 4,066(52.52%) |  |
| **Daily energy intake (kCal)** | 2,072.59±602.88 | 2,058.73±600.06 | 2,289.57±605.92 | <0.001 | 2,061.43±624.85 | 2,073.82±600.44 | 0.378 |
| **PhenoAge** | -5.47±4.26 | -5.21±4.21 | -9.58±2.64 | <0.001 | 2.59±3.23 | -6.36±3.32 | <0.001 |
| **KDM Age** | 17.16±10.25 | 18.57±8.80 | -4.91±4.13 | <0.001 | 27.22±8.38 | 16.06±9.82 | <0.001 |
| **AHEI** | 31.17±6.63 | 31.08±6.63 | 32.64±6.40 | <0.001 | 30.69±6.60 | 31.22±6.63 | 0.055 |
| **aMED** | 2.16±1.18 | 2.16±1.17 | 2.23±1.22 | 0.132 | 2.08±1.12 | 2.17±1.18 | 0.068 |
| **DASH** | 18.36±4.14 | 18.33±4.11 | 18.77±4.58 | 0.056 | 18.07±4.11 | 18.39±4.15 | 0.079 |
| **DII** | 0.92±1.76 | 0.94±1.76 | 0.52±1.66 | <0.001 | 1.12±1.76 | 0.89±1.76 | <0.001 |
| **HEI2020** | 30.13±7.29 | 30.19±7.30 | 29.15±7.00 | 0.005 | 30.50±7.52 | 30.08±7.26 | 0.240 |
| 1 mean ± sd for continuous; n (%) for categorical | |  |  |  |  |  |  |
| 2 Wilcoxon rank sum test; Pearson’s Chi-squared test with simulated p-value | | |  |  |  |  |  |
|  | |  |  |  |  |  |  |

# **Supplementary Table 2.** Linear regression analysis in UK Biobank

|  | **Model 1^a^** | | | | | | **Model 2^b^** | | | | | | **Model 3^c^** | | | | | |
| --- | --- | --- | --- | --- | --- | --- | --- | --- | --- | --- | --- | --- | --- | --- | --- | --- | --- | --- |
|  | **PhenoAge** | | | **KDM Age** | | | **PhenoAge** | | | **KDM Age** | | | **PhenoAge** | | | **KDM Age** | | |
|  | **β** | **95% CI** | ***P Value*** | **β** | **95% CI** | ***P Value*** | **β** | **95% CI** | ***P Value*** | **β** | **95% CI** | ***P Value*** | **β** | **95% CI** | ***P Value*** | **β** | **95% CI** | ***P Value*** |
| **AHEI** | -0.03 | -0.04, -0.01 | **<0.001** | -0.12 | -0.15, -0.09 | **<0.001** | -0.03 | -0.04, -0.01 | **<0.001** | -0.12 | -0.15, -0.09 | **<0.001** | -0.02 | -0.03, -0.01 | **0.005** | -0.10 | -0.13, -0.07 | **<0.001** |
| **DASH** | -0.04 | -0.07, -0.02 | **<0.001** | -0.19 | -0.24, -0.15 | **<0.001** | -0.05 | -0.07, -0.03 | **<0.001** | -0.19 | -0.24, -0.14 | **<0.001** | -0.04 | -0.06, -0.02 | **<0.001** | -0.17 | -0.21, -0.12 | **<0.001** |
| **aMED** | -0.14 | -0.22, -0.06 | **<0.001** | -0.55 | -0.71, -0.38 | **<0.001** | -0.14 | -0.22, -0.07 | **<0.001** | -0.52 | -0.69, -0.35 | **<0.001** | -0.08 | -0.15, -0.01 | **0.033** | -0.39 | -0.55, -0.23 | **<0.001** |
| **HEI2020** | 0.01 | 0.00, 0.02 | 0.102 | 0.02 | -0.01, 0.04 | 0.246 | 0.01 | -0.01, 0.02 | 0.263 | 0.02 | -0.01, 0.04 | 0.197 | 0.00 | -0.01, 0.01 | 0.849 | 0.00 | -0.02, 0.03 | 0.834 |
| **DII** | 0.14 | 0.09, 0.19 | **<0.001** | 0.49 | 0.38, 0.60 | **<0.001** | 0.13 | 0.08, 0.18 | **<0.001** | 0.47 | 0.36, 0.58 | **<0.001** | 0.10 | 0.05, 0.15 | **<0.001** | 0.41 | 0.30, 0.52 | **<0.001** |
| Abbreviation: CI = Confidence Interval  Model 1^a^: adjusting age, sex and race  Model 2^b^: model 1**+**additional adjusting family size, education level, deprivation index and marital status  Model 3^c^: model 2**+**additional adjusting BMI, smoking status, alcohol consumption, physical activity and history of hypertension | | | | | | | | | | | | | | | | | | |

# **Supplementary Table 3.** Quartile regression and linear trend analysis in UK Biobank

|  | **PhenoAge** | | | **KDM Age** | | |
| --- | --- | --- | --- | --- | --- | --- |
|  | **β** | **95% CI** | ***P Value*** | **β** | **95% CI** | ***P Value*** |
| **AHEI** |  |  |  |  |  |  |
| Q1 | Reference | Reference |  | Reference | Reference |  |
| Q2 | -0.25 | -0.49, -0.02 | **0.035** | -0.75 | -1.30, -0.23 | **0.005** |
| Q3 | -0.31 | -0.55, -0.08 | **0.009** | -1.20 | -1.70, -0.64 | **<0.001** |
| Q4 | -0.33 | -0.56, -0.09 | **0.007** | -1.70 | -2.20, -1.20 | **<0.001** |
| P for linear |  |  | **0.007** |  |  | **<0.001** |
| **aMED** |  |  |  |  |  |  |
| Q1 | Reference | Reference |  | Reference | Reference |  |
| Q2 | -0.09 | -0.33, 0.14 | 0.437 | -0.24 | -0.76, 0.28 | 0.371 |
| Q3 | -0.08 | -0.31, 0.16 | 0.526 | -0.89 | -1.40, -0.37 | **<0.001** |
| Q4 | -0.29 | -0.53, -0.05 | **0.018** | -1.20 | -1.70, -0.67 | **<0.001** |
| P for linear |  |  | **0.027** |  |  | **<0.001** |
| **DASH** |  |  |  |  |  |  |
| Q1 | Reference | Reference |  | Reference | Reference |  |
| Q2 | -0.14 | -0.38, 0.09 | 0.237 | -0.81 | -1.30, -0.29 | **0.002** |
| Q3 | -0.25 | -0.49, -0.01 | **0.038** | -1.10 | -1.60, -0.59 | **<0.001** |
| Q4 | -0.36 | -0.60, -0.12 | **0.003** | -1.80 | -2.30, -1.30 | **<0.001** |
| P for linear |  |  | **0.002** |  |  | **<0.001** |
| **HEI2020** |  |  |  |  |  |  |
| Q1 | Reference | Reference |  | Reference | Reference |  |
| Q2 | 0.09 | -0.14, 0.33 | 0.434 | 0.46 | -0.06, 0.99 | 0.081 |
| Q3 | -0.03 | -0.27, 0.21 | 0.795 | -0.17 | -0.70, 0.35 | 0.514 |
| Q4 | -0.03 | -0.26, 0.21 | 0.822 | 0.05 | -0.47, 0.58 | 0.845 |
| P for linear |  |  | 0.587 |  |  | 0.567 |
| **DII** |  |  |  |  |  |  |
| Q1 | Reference | Reference |  | Reference | Reference |  |
| Q2 | 0.05 | -0.18, 0.29 | 0.653 | 0.43 | -0.09, 0.95 | 0.106 |
| Q3 | 0.21 | -0.03, 0.45 | 0.080 | 1.10 | 0.61, 1.70 | **<0.001** |
| Q4 | 0.46 | 0.22, 0.70 | **<0.001** | 1.80 | 1.30, 2.30 | **<0.001** |
| P for linear |  |  | **<0.001** |  |  | **<0.001** |
| Abbreviation: CI = Confidence Interval | |  |  |  |  |  |
|  | | |  |  |  |  |

| Model | NHANES (Training Set) | | | | | UK Biobank (Validation Set) | | | | |
| --- | --- | --- | --- | --- | --- | --- | --- | --- | --- | --- |
|  | Accuracy | Sensitivity | Specificity | F1 Score | AUC | Accuracy | Sensitivity | Specificity | F1 Score | AUC |
| Gradient Boosting Decision Tree (GBDT) | 0.676 | 0.576 | 0.727 | 0.543 | 0.711 | 0.742 | 0.755 | 0.516 | 0.847 | 0.706 |
| Random Forest (RF) | 0.664 | 0.558 | 0.716 | 0.526 | 0.688 | 0.862 | 0.893 | 0.316 | 0.924 | 0.720 |
| Decision Tree (DT) | 0.648 | 0.587 | 0.678 | 0.527 | 0.672 | 0.747 | 0.763 | 0.456 | 0.851 | 0.629 |
| Tabular Prior-Data Fitted Networks (TabPFN) | 0.657 | 0.564 | 0.704 | 0.524 | 0.688 | 0.896 | 0.934 | 0.215 | 0.944 | 0.757 |
| Logistic Regression (LR) | 0.630 | 0.563 | 0.664 | 0.505 | 0.658 | 0.945 | 0.996 | 0.028 | 0.971 | 0.639 |
| Light Gradient Boosting Machine (LightGBM) | 0.673 | 0.569 | 0.726 | 0.538 | 0.700 | 0.877 | 0.912 | 0.238 | 0.933 | 0.705 |
| Categorical Boosting (CatBoost) | 0.686 | 0.585 | 0.737 | 0.555 | 0.729 | 0.864 | 0.901 | 0.196 | 0.926 | 0.690 |
| Deep Learning (DL) | 0.671 | 0.635 | 0.690 | 0.564 | 0.719 | 0.074 | 0.022 | 0.993 | 0.043 | 0.670 |
| XGBoost | 0.659 | 0.565 | 0.706 | 0.525 | 0.687 | 0.898 | 0.939 | 0.173 | 0.946 | 0.686 |

# **Supplementary Table 4.** Comparison of discrimination characteristics among different models

# **Supplementary Table 5.** Hyperparameter values of models

| Models | Hyperparameters | Values |
| --- | --- | --- |
| Random Forest (RF) | Number of trees | 287 |
|  | Maximum tree depth | 9 |
|  | The minimum number of samples for internal node splitting | 191 |
|  | The minimum sample size of leaf nodes | 59 |
| Decision Tree (DT) | Maximum tree depth | 9 |
|  | The minimum number of samples for internal node splitting | 183 |
|  | The minimum sample size of leaf nodes | 200 |
| Logistic Regression (LR) | Solver | liblinear |
|  | Penalty | L2 |
|  | C | 22.79 |
| Light Gradient Boosting Machine (LightGBM) | Maximum tree depth | 7 |
|  | The minimum sample size of leaf nodes | 48 |
|  | Maximum number of leaves | 16 |
| Categorical Boosting (CatBoost) | Learning rate | 0.1 |
|  | Number of iterations | 61 |
|  | Maximum tree depth | 8 |
|  | The minimum sample size of leaf nodes | 52 |
| Deep Learning (DL) | Units in layer 1 | 25 |
|  | Dropout ratio of layer 1 | 0.25 |
|  | Units in layer 2 | 10 |
|  | Dropout ratio of layer 2 | 0.25 |
|  | Units in layer 3 | 1 |
|  | Learning rate | 0.001 |
|  | Training rounds | 40 |
|  | Batch size | 500 |
| XGBoost | Learning rate | 0.1 |
|  | Number of trees | 77 |
|  | Maximum tree depth | 3 |
|  | Minimum weight sum of child nodes | 27 |

# **Supplementary reference**

[1] KRESOVICH J K, PARK Y-M M, KELLER J A, et al. Healthy eating patterns and epigenetic measures of biological age [J]. Am J Clin Nutr, 2022, 115(1): 171-9.

[2] CHIUVE S E, FUNG T T, RIMM E B, et al. Alternative dietary indices both strongly predict risk of chronic disease [J]. J Nutr, 2012, 142(6): 1009-18.

[3] ZHAN J J, HODGE R A, DUNLOP A L, et al. Dietaryindex: A User-Friendly and Versatile R Package for Standardizing Dietary Pattern Analysis in Epidemiological and Clinical Studies [J]. BioRxiv : the Preprint Server For Biology, 2023.

[4] JAYANAMA K, THEOU O, GODIN J, et al. Relationship between diet quality scores and the risk of frailty and mortality in adults across a wide age spectrum [J]. BMC Med, 2021, 19(1): 64.

[5] TIAN T, ZHANG J, XIE W, et al. Dietary Quality and Relationships with Metabolic Dysfunction-Associated Fatty Liver Disease (MAFLD) among United States Adults, Results from NHANES 2017-2018 [J]. Nutrients, 2022, 14(21).

[6] KREBS-SMITH S M, PANNUCCI T E, SUBAR A F, et al. Update of the Healthy Eating Index: HEI-2015 [J]. J Acad Nutr Diet, 2018, 118(9): 1591-602.

[7] MARX W, VERONESE N, KELLY J T, et al. The Dietary Inflammatory Index and Human Health: An Umbrella Review of Meta-Analyses of Observational Studies [J]. Adv Nutr, 2021, 12(5): 1681-90.

[8] JURADO-FASOLI L, CASTILLO M J, AMARO-GAHETE F J. Dietary Inflammatory Index and S-Klotho Plasma Levels in Middle-Aged Adults [J]. Nutrients, 2020, 12(2).

[9] SHIVAPPA N, STECK S E, HURLEY T G, et al. Designing and developing a literature-derived, population-based dietary inflammatory index [J]. Public Health Nutr, 2014, 17(8): 1689-96.

[10] FUNG T T, REXRODE K M, MANTZOROS C S, et al. Mediterranean diet and incidence of and mortality from coronary heart disease and stroke in women [J]. Circulation, 2009, 119(8): 1093-100.
